# Heterotrimeric G-alpha subunits Gpa11 and Gpa12 define a transduction pathway that control spore size and virulence in *Mucor circinelloides*

**DOI:** 10.1371/journal.pone.0226682

**Published:** 2019-12-30

**Authors:** J. Alberto Patiño-Medina, Nancy Y. Reyes-Mares, Marco I. Valle-Maldonado, Irvin E. Jácome-Galarza, Carlos Pérez-Arques, Rosa E. Nuñez-Anita, Jesús Campos-García, Verónica Anaya-Martínez, Rafael Ortiz-Alvarado, Martha I. Ramírez-Díaz, Soo Chan Lee, Victoriano Garre, Víctor Meza-Carmen

**Affiliations:** 1 Instituto de Investigaciones Químico Biológicas, Universidad Michoacana de San Nicolás de Hidalgo (UMSNH), Morelia, Michoacán, México; 2 Departamento de Biología Molecular, Laboratorio Estatal de Salud Pública del Estado de Michoacán, Morelia, Michoacán, México; 3 Departamento de Genética y Microbiología, Facultad de Biología, Universidad de Murcia, Murcia, España; 4 Facultad de Medicina Veterinaria y Zootecnia, Universidad Michoacana de San Nicolás Hidalgo, Morelia, Michoacán, Mexico; 5 Facultad de Ciencias de la Salud, Universidad Anáhuac, Naucalpan de Juarez, Estado de México, México; 6 Facultad de Químico Farmacobiología, Universidad Michoacana de San Nicolás de Hidalgo, Morelia, Michoacan, México; 7 Department of Biology, South Texas Center of Emerging Infectious Diseases (STCEID), University of Texas at San Antonio, San Antonio, Texas, United States of America; Soonchunhyang University, REPUBLIC OF KOREA

## Abstract

*Mucor circinelloides* is one of the causal agents of mucormycosis, an emerging and high mortality rate fungal infection produced by asexual spores (sporangiospores) of fungi that belong to the order Mucorales. *M*. *circinelloides* has served as a model genetic system to understand the virulence mechanism of this infection. Although the G-protein signaling cascade plays crucial roles in virulence in many pathogenic fungi, its roles in Mucorales are yet to be elucidated. Previous study found that sporangiospore size and calcineurin are related to the virulence in *Mucor*, in which larger spores are more virulent in an animal mucormycosis model and loss of a calcineurin A catalytic subunit CnaA results in larger spore production and virulent phenotype. The *M*. *circinelloides* genome is known to harbor twelve *gpa* (*gpa1* to *gpa12*) encoding G-protein alpha subunits and the transcripts of the *gpa11* and *gpa12* comprise nearly 72% of all twelve *gpa* genes transcript in spores. In this study we demonstrated that loss of function of Gpa11 and Gpa12 led to larger spore size associated with reduced activation of the calcineurin pathway. Interestingly, we found lower levels of the *cnaA* mRNAs in sporangiospores from the Δ*gpa12* and double Δ*gpa11*/Δ*gpa12* mutant strains compared to wild-type and the Δ*cnaA* mutant had significantly lower *gpa11* and *gpa12* mRNA levels compared to wild-type. However, in contrast to the high virulence showed by the large spores of Δ*cnaA*, the spores from Δ*gpa11*/Δ*gpa12* were avirulent and produced lower tissue invasion and cellular damage, suggesting that the *gpa11* and *gpa12* define a signal pathway with two branches. One of the branches controls spore size through regulation of calcineurin pathway, whereas virulences is controlled by an independent pathway. This virulence-related regulatory pathway could control the expression of genes involved in cellular responses important for virulence, since sporangiospores of Δ*gpa11*/Δ*gpa12* were less resistant to oxidative stress and phagocytosis by macrophages than the Δ*cnaA* and wild-type strains. The characterization of this pathway could contribute to decipher the signals and mechanism used by Mucorales to produce mucormycosis.

## Introduction

*Mucor circinelloides* is a basal fungus that belongs to the Phylum Zygomycota [1]. This organism produces three types of spores, zygospores are produced by mating of hyphae of opposite sex: (+) and (-). Arthrospores are generated by hyphae in submerged cultures after the end of the exponential growth or under unfavorable growth conditions. Sporangiospores are produced by asexual reproduction on solid substrate in large number [2, 3]. Several growth conditions determine whether *M*. *circinelloides* sporangiospores (spores) will produce mycelium or yeast; the most well-studied being the presence of carbon source and atmospheric conditions during spore germination [4–6]. *M*. *circinelloides* is one of the etiological agents of the mucormycosis, a fungal infection that affects mainly immunocompromised patients [7–8]. This infection has called medical attention as its incidence has risen worldwide recently [9–11], and relatively high mortality rates have been reported [12–13]. In this sense, the fate of vegetative cells in *M*. *circinelloides* has implications for infectiveness, as the mycelial stage usually shows virulent morphology [14–15], while the yeast morphology is less virulent [15]. Furthermore, the ability to germinate of the Mucorales sporangiospores in the immunocompromised patient is a critical step for stablishing the infection [12, 16].

Larger spores lead to a faster germination rate that, in turn, correlates with a more virulent phenotype in *M*. *circinelloides* [17]. Spore size in *M*. *circinelloides* is controlled in part by CnaA, which is the catalytic subunit A of the phosphatase Ca^+2^-dependent calcineurin [15]. We have previously described that ADP-ribosylation factor-1 (Arf1) dysfunction also increases spore size and virulence and generates defects during the aerobic growth in *M*. *circinelloides* [18]. Moreover, additional molecular regulators of dimorphism in *M*. *circinelloides* have been reported. Thus, deletion of the calcineurin regulatory subunit CnbB-encoding gene promotes the yeast rather than mycelial form, even in the presence of oxygen, and this mutant is less virulent compared to its wild-type form [15]. Additionally, deletion of *arf2* leads to difficulty in the generation of the yeast morphology [18]. In fungi, the protein kinase A (PKA) pathway is a key signaling pathway that controls spore production, spore germination, resistance to different stress conditions, and virulence [19]; deletion of *pkaR1*, which encodes a regulatory subunit 1 of PKA, leads to a decrease in spore production and shorter hyphae length after yeast-mycelium transition in *M*. *circinelloides* [20].

Heterotrimeric G proteins are regulators of the PKA pathway, and their canonical functions are involved in the control of the activity of several effectors; for example, adenylyl cyclase, lipid modifying enzymes like phospholipase C, and ion channels [21–22]. In the ascomycetes *Aspergillus nidulans* and *A*. *parasiticus*, the Gα subunit FadA stimulates the cAMP levels leading to the activation of the PkaC subunit, which ultimately controls the gene transcription involved in sporulation [23–24].

Our group previously reported that the largest number of members of genes encoding heterotrimeric G proteins in the Fungi kingdom is present in *M*. *circinelloides* [25]. The twelve Gα-encoding genes (*gpa*1-12) were grouped into three (I-III) of the four groups of the Gα fungal phylogenetic classification [25–26]. In *M*. *circinelloides*, Gpa11 and Gpa12, which belong to group I (Gα_s_) and group III (Gα_i_), respectively, showed increased transcript levels during the spore stage (~69% of the total *gpa* transcript levels), suggesting that these genes have an important role in spore production or germination in this Mucoral [25]. However, the molecular role of these subunits in spore function is still unknown.

The aim of this work was to functionally characterize the role of the heterotrimeric Gα subunits Gpa11 and Gpa12 in sporulation and vegetative growth and determine their role in the virulence of *M*. *circinelloides*.

## Results

### Deletion of *gpa11* and *gpa12* in *M*. *circinelloides*

**Fig 1:**
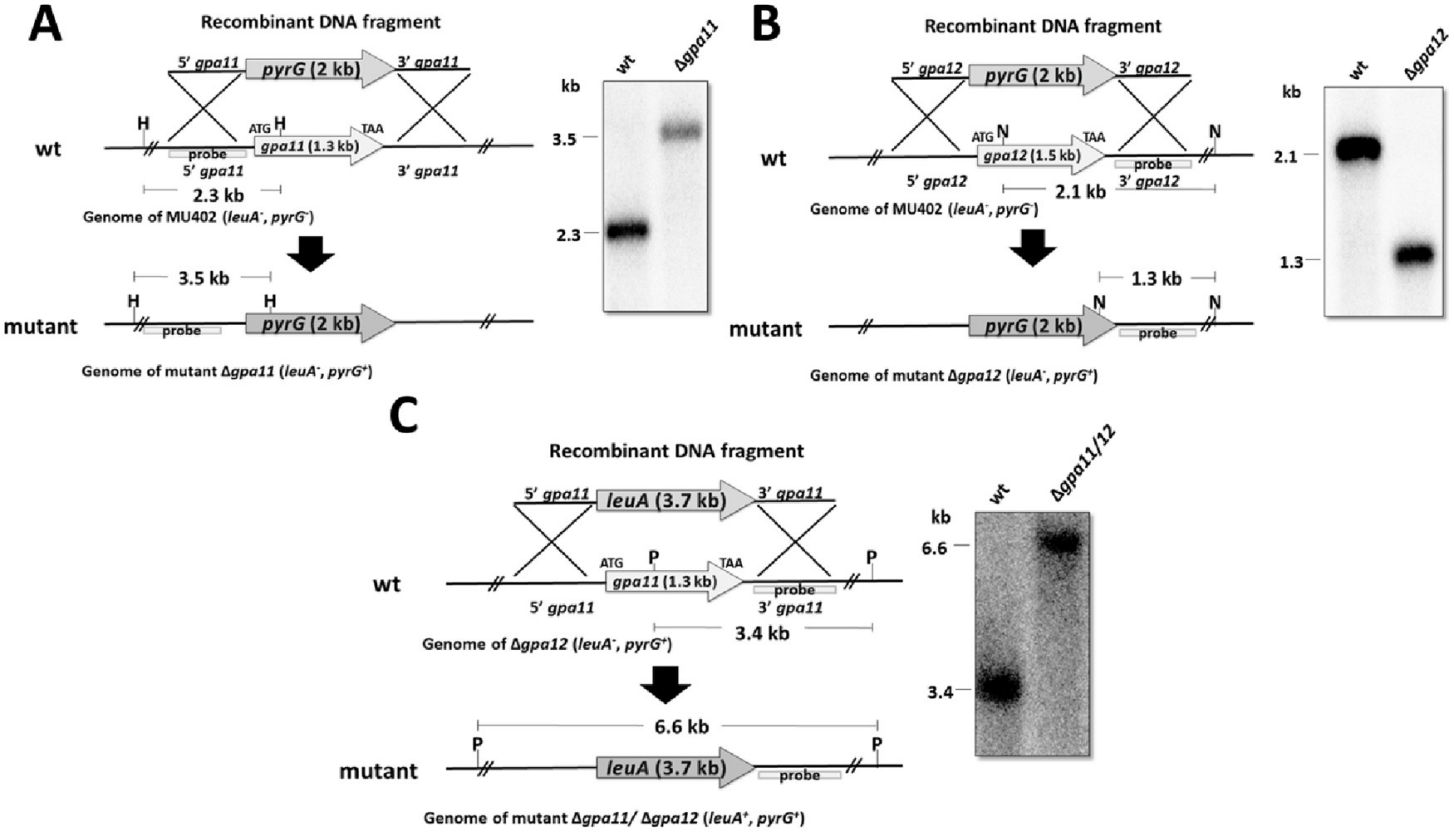
Deletion of *gpa11* and *gpa12* in *M*. *circinelloides* confirmed by Southern-blot. **A)** *gpa11*; **B)** *gpa12*; and **C)** *gpa11/gpa12* deletion. The 5′ and 3′ regions (1.1 kb for each) upstream and downstream from the start and stop codons, respectively, of *gpa11* or *gpa12* were designed to flank the *pyrG* selection marker for gene deletion (**A** and **B**). For the deletion of *gpa11* in Δ*gpa12*, the 5′ and 3′ regions (1.1 kb for each) upstream and downstream from the start and stop codons, respectively, of *gpa11* were designed to flank *leuA* (**C**). For each diagram, the recombinant fragments that were used to transform protoplasts of the strain MU402 (*pyrG*^−^, *leuA*^−^) are shown. Molecular confirmation by Southern blotting was performed using specific probes for each *gpa* gene. DNA samples from transformed and parental (MU402) strains were digested with the indicated restriction enzymes (H, *Hin*cII; N, *Nco*I; P, *Pvu*II).

*M*. *circinelloides* exhibits twelve Gα subunits (Gpa1-Gpa12). The mRNA of two of these subunits, Gpa11 and Gpa12, have been reported to accumulate mainly in the spore stage and decrease during vegetative growth [25]. To determine whether these genes are relevant to spore physiology, we created *gpa11 and gpa12* deletion mutants. Replacement fragments of *gpa11* and *gpa12* that contained 5′ upstream and 3′ downstream regions from *gpa11* or *gpa12* flanking the selective marker *pyrG* were used to transform protoplasts of the *M*. *circinelloides* wild-type strain MU402 (*leuA*^-^, *pyrG*^-^), which is auxotrophic for leucine and uracil [27], to generate *gpa11* and *gpa12* deletion strains (Fig 1). Transformants grown in selective medium (MMC) were recovered and subsequent vegetative cycles in selective medium were performed to obtain homokaryotic Δ*gpa11* and Δ*gpa12* mutants. After 5 cycles of vegetative growth/sporulation, we obtained two independent transformants for *gpa11* and four for *gpa12* that showed 100% Ura^+^ spores, confirming the homokaryosis.

The presence of 2.3-kb and 3.5-kb hybridization fragments in wild-type and Δ*gpa11*, respectively; confirmed the presence of a unique integration event in the desired locus, and homokaryosis of the *gpa11* deletant (Fig 1A). Meanwhile, the presence of 2.1-kb and 1.3-kb hybridization fragments in wild-type and *gpa12*, respectively revealed the mutation in *gpa12* (Fig 1B).

A mutant lacking both genes was generated by transformation of the Δ*gpa12* strain with a replacement fragment containing the selective marker *leuA* flanked by 5′ upstream and 3′ downstream regions of *gpa11*. Transformants grown in selective media (without uracil and leucine) were characterized by Southern blot hybridization. A 6.6-kb hybridization band in the selected transformant confirmed the presence of a unique integration event in the desired locus and the homokaryosis of the double Δ*gpa11*/Δ*gpa12* knockout mutant (Fig 1C).

**Fig 2:**
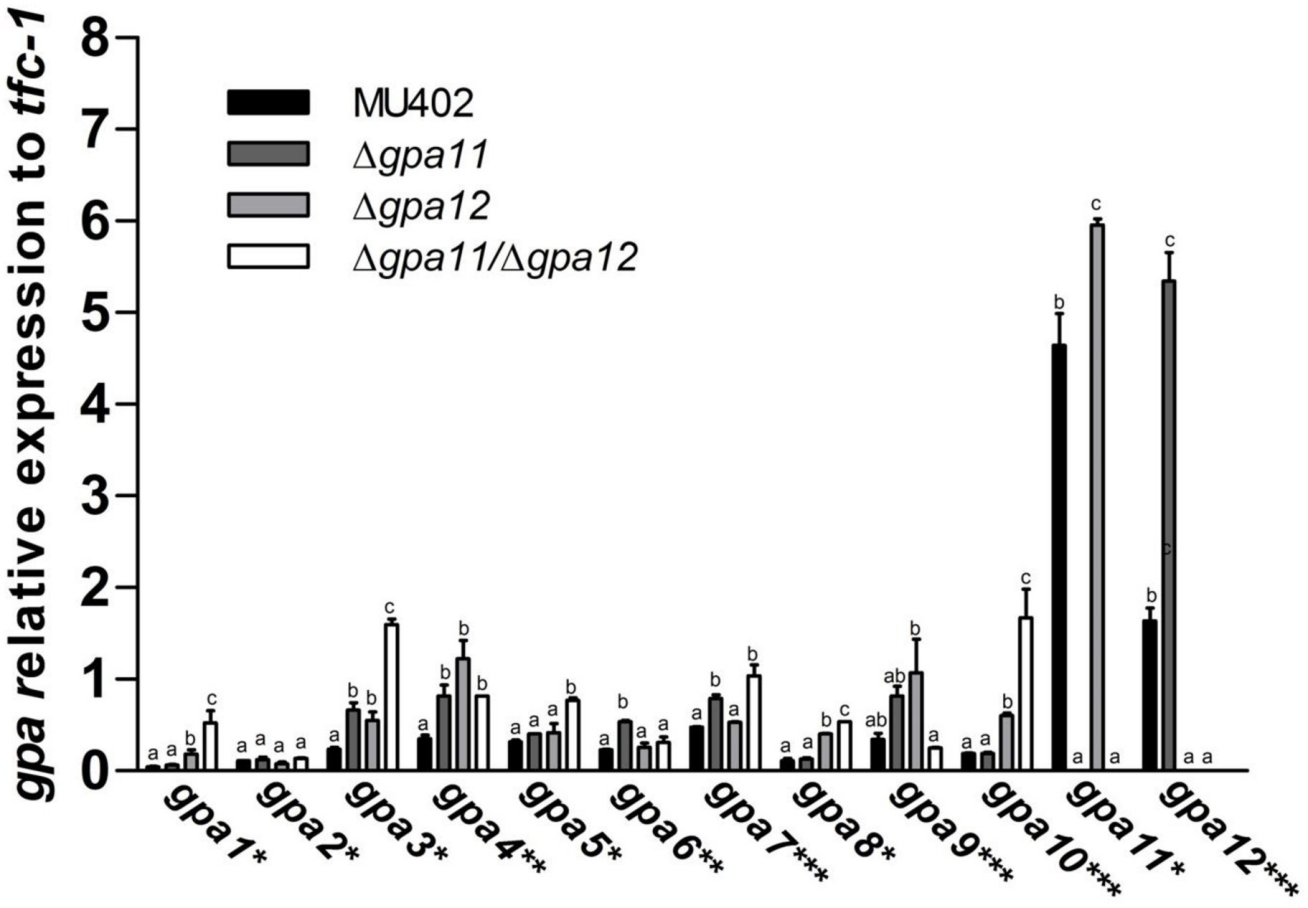
mRNA quantitation of *gpa* genes in sporangiospores in *gpa11* and *gpa12* deletion mutants of *M*. *circinelloides*. Total RNA was isolated from spores of the wild-type, Δ*gpa11*, Δ*gpa12*, and Δ*gpa11*/Δ*gpa12*. The mRNA from each *gpa* gene was determined by qRT-PCR. Asterisks denote the phylogenetic group of the Gpa proteins in *M*. *circinelloides* (* I; **-II, and ***-III) [25]. Four independent experiments were performed under the same conditions. Different letters denote statistically significant differences (ANOVA, Fisher, p<0.05).

Expression analysis of *gpa11* and *gpa12* in spores generated from single and double knockout mutants confirmed the null expression of those genes (Fig 2).

For the further analysis, we used two independent strains for each gene mutation revealing similar results for these independent strains in all experiment tested. Furthermore, single Δ*gpa11* and Δ*gpa12* mutant strains were complemented with their respective wild-type gene by expression in the pEUKA4 vector [28] generating the Δ*gpa11+gpa11*wt and Δ*gpa12+gpa12*wt strains.

Interestingly, the single mutation of each *gpa* gene provoked a significant upregulation of the other. Thus, deletion of *gpa11* led to an ~30% increase in *gpa12* mRNA levels, while deletion of *gpa12* resulted in an ~226% increase of *gpa11* mRNA levels, suggesting a compensatory mechanism (Fig 2, S1 Table). Moreover, single deletion of *gpa11* and *gpa12* led to a significant increase in the mRNA levels of several *gpa* genes. The expression of some *gpa* genes was increased more than two folds in Δ*gpa11* (*gpa3*, *gpa4*, *gpa6*, *gpa9*, and *gpa12*), and in Δ*gpa12* (*gpa1*, *gpa3*, *gpa4*, *gpa8*, *gpa9*, and *gpa10*) (Fig 2, S1 Table). In addition, deletion of both genes led to a further rise in mRNA levels of some *gpa* genes, including *gpa1* (~11.73 folds), *gpa3* (~6.8 folds), and *gpa8* (~4.78 folds), which encode proteins that belong to group I of the Gα phylogenetic fungal classification (Gα_s_). Also, the mRNA levels of a gene, *gpa10*, encoding a protein belonging to group III (Gα_I_) of the phylogenetic fungal classification was highly increased (~8.77 folds) in the double-*gpa* mutant strain (Fig 2, S1 Table).

### Gpa11 and Gpa12 are involved in sporangiospore size and germination rate in *M*. *circinelloides*

**Fig 3:**
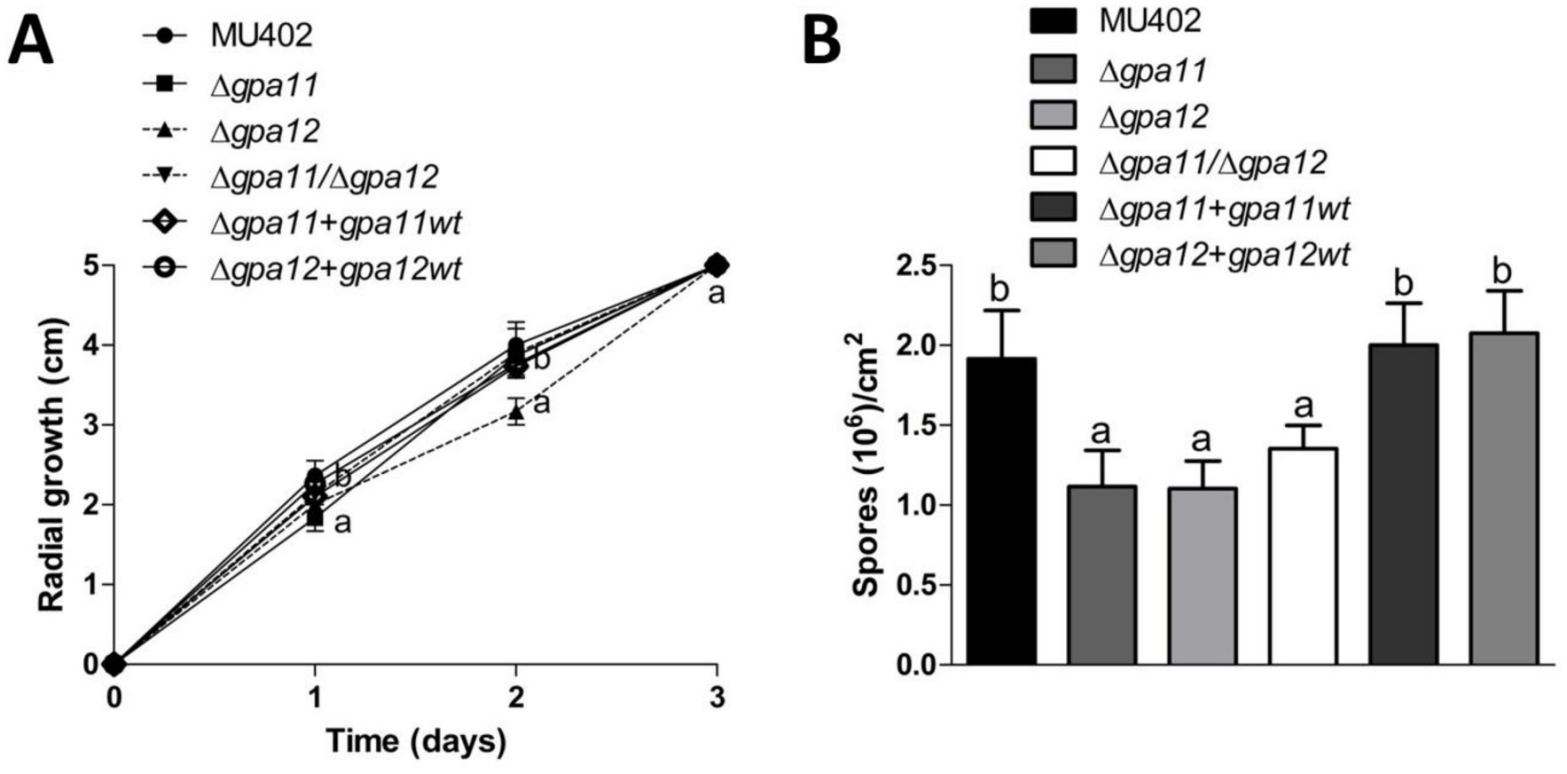
Sporangiospore production in Δ*gpa11* and Δ*gpa12* mutants of *M*. *circinelloides*. 50 spores were grown in YPG agar plates and **A)** radial growth was recorded each day during the experiment. **B)** Spores grown after 5 days were counted. Four independent experiments were performed for each condition. Statistically significant differences are indicated by different letters (ANOVA and Fisher’s tests; *p* ≤ 0.05).

In order to investigate the physiological role of *gpa11* and *gpa12*, radial growth and spore production of the different strains were assessed. The radial growth of all the mutant strains was similar to the wild-type strain at the end of the experiment (Fig 3A). After 5 days of incubation, there was around 42–46% decrease in spore production in all single and double mutant strains compared to the wild-type strain (Fig 3B), indicating that both genes participate in spore production. Additionally, the Δ*gpa12* strain produced slightly less spores compared to other mutant strain. The Δ*gpa11+gpa11*wt and Δ*gpa12+gpa12*wt complemented strains did not show significant difference in spore production compared to the wild-type strain.

**Fig 4:**
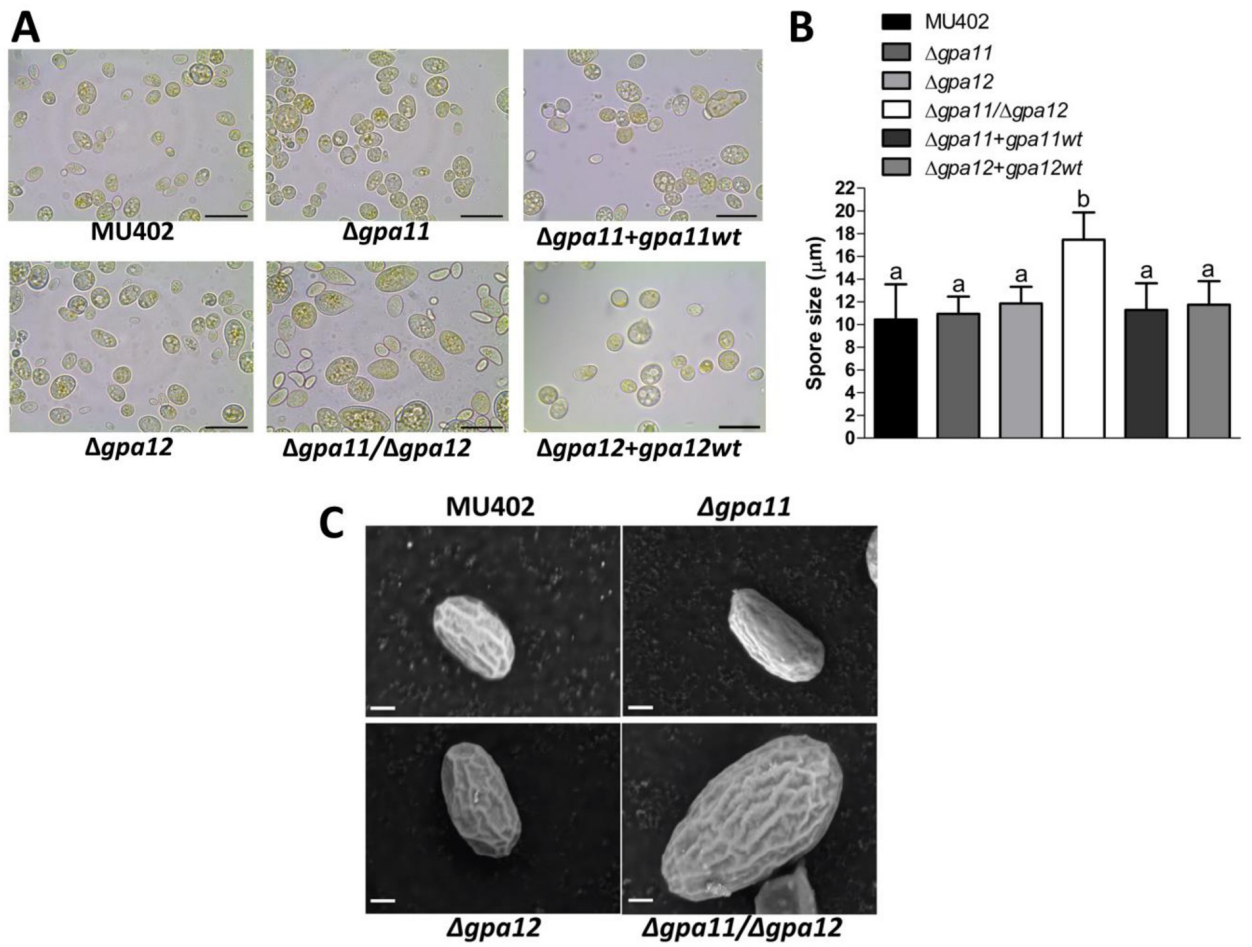
Influence of *gpa11* and *gpa12* on sporangiospore size in *M*. *circinelloides*. The spores from the different *M*. *circinelloides* knockout strains produced in YPG were observed under **A)** light microscope (100 X), scale bar equal to 20 μm. **B)** The spore size of each strain was quantified using the Leica Application Suite. **C)** The spores obtained after five days of incubation on solid YPG media were observed under scanning electron microscope. Representative photographs from the corresponding strains of *M*. *circinelloides* under 10,000 magnifications. Scale bar is equal to 1 μm. Statistically significant differences are indicated by different letters (ANOVA and Fisher’s tests; *p* ≤ 0.05).

The spores produced on YPG media were observed by light microscopy. The spores from the Δ*gpa11* mutant strain were morphologically similar (in size) to those produced from the wild-type strain (on average 10.3 and 10.2 μm, respectively) (Fig 4). While the Δ*gpa12* spores were slightly larger than those of the wild-type strain, at 11.4 μm diameter on average. Interestingly, the Δ*gpa11*/Δ*gpa12* mutant strain generated the largest spore size, compared to the rest of the strains, at 16.3 μm diameter on average (Fig 4), but some of them were up to 20 μm of size. Interestingly, the Δ*gpa11+gpa11*wt and Δ*gpa12+gpa12*wt complemented strains showed similar size as the wild-type strain (Fig 4A and 4B). The spore size was also evaluated by scanning electron microscopy, confirming that the spores from the Δ*gpa11*/Δ*gpa12* mutant were larger than those of the other strains and all had a similar surface shape (Fig 4C). Similar results in terms of spore size were observed with independent *gpa* mutant strains (S1 Fig).

**Fig 5:**
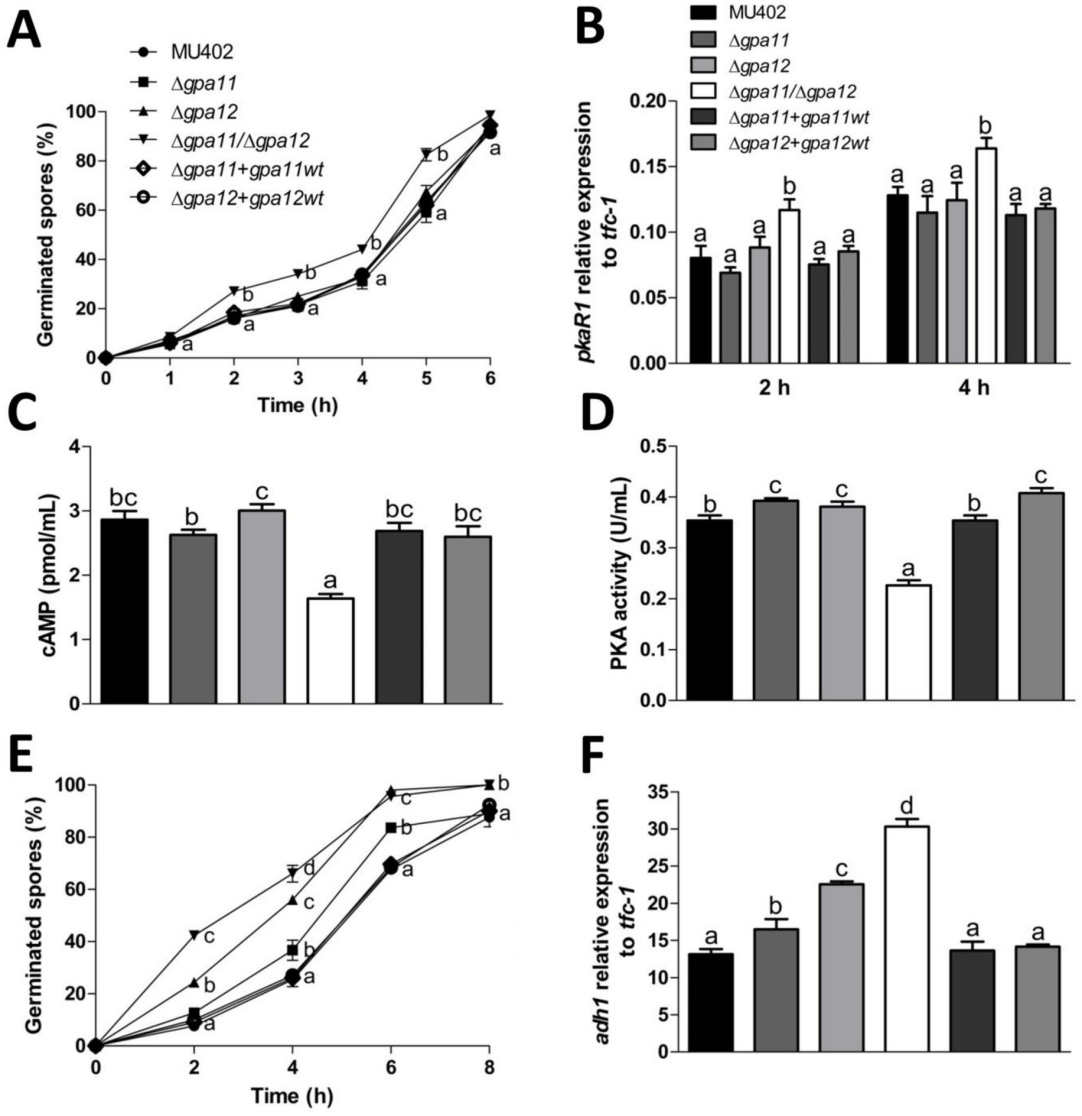
Sporangiospore germination of *M*. *circinelloides gpa11* and *gpa12* deletion mutants. **A)** Percentage of germinated spores in aerobic conditions from each strain in YPG. Aerobic germinated cells were considered independent of hyphae length or number of mother cells. **B)** Relative expression of *pkaR1*, quantified by qRT-PCR using *tfc-1* as reference gene from hyphae obtained from each strain grown at 2 and 4 h. ΔCT analysis was performed to compare the mRNA levels between strains. Spores from the different strains from *M*. *circinelloides* were inoculated into YPG media and incubated with constant shaking (150 rpm) for 3 h, and **C)** intracellular cAMP levels, and **D)** PKA activity were determined. **E)** Percentage of spores germinated under anaerobic conditions in YPG media. **F)** Relative expression of *adh1* determined from yeast cells at 4 h of growth, ΔCT analysis was performed to compare mRNA levels between strains. Four independent experiments were performed for each condition. Statistically significant differences are indicated by different letters (ANOVA and Fisher’s tests; *p* ≤ 0.05).

It has been described in *M*. *circinelloides* that an increase in spore size correlates with a faster aerobic germination rate [17]. To confirm this assumption, we measured the germination rate of the different strains grown in YPG. The double *gpa*-mutant strain had a 50% higher germination rate after 2 h of growth compared to the wild-type strain (Fig 5A). However, both single *gpa*-mutant and complemented strains germinated at very similar rates to the wild-type strain (Fig 5A).

Aerobic germination was corroborated using the gene marker *pkaR1*, which is a gene expressed preferentially during hyphae growth compared to the yeast or spore stage [29]. Only the Δ*gpa11*/Δ*gpa12* mutant strain accumulated significantly more *pkaR1* mRNA observed in the germinating spores after 2 (40%) and 4 h (28%) in YPG with respect to other strains (Fig 5B). The higher mRNA levels of *pkaR1* in the Δ*gpa11*/Δ*gpa12* mutant strain suggest a repression in the PKA pathway. To probe this hypothesis, we conducted the quantification of cAMP levels and PKA activity in the different strains after 3 h of aerobic germination. In correlation with the mRNA levels, the only strain that showed lower cAMP levels and PKA activity was the Δ*gpa11*/Δ*gpa12* mutant strain, meanwhile the rest of the strains showed similar levels as the wild-type strain (Fig 5C and 5D).

Germination rate under anaerobic conditions revealed that Δ*gpa12* and Δ*gpa11*/Δ*gpa12* mutants germinated faster than the Δ*gpa12*, complemented and wild-type strains. The double *gpa*-mutant strain demonstrated a higher rate of germination (5.52 folds) after 2 h compared to the wild-type strain (Fig 5E). The mRNA levels of *adh1*, which is preferentially expressed during anaerobic growth [29–30], were significantly higher in Δ*gpa11* (21%), Δ*gpa12* (71%) and Δ*gpa11*/Δ*gpa12* (130%) strains after 4 h of growth compared to the wild-type strain (Fig 5F).

These results indicated that the products of *gpa11* and *gpa12* genes are involved in the control of the regulation of sporangiospore size as well as the aerobic and anaerobic germination rates in *M*. *circinelloides*.

### Mutation of *gpa12* and the double mutation of *gpa11* and *gpa12* decreased *cnaA* mRNA levels in sporangiospores in *M*. *circinelloides*

It has been reported that dysfunction of the calcineurin catalytic subunit A (CnaA) leads to an increase in the spore size of *M*. *circinelloides* [15]. Based on the larger spore phenotype observed in the Δ*gpa11*/Δ*gpa12* mutant strain, we performed mRNA quantitation by qRT-PCR of *cnaA* in spores and during aerobic germination from *gpa*-mutant strains.

**Fig 6:**
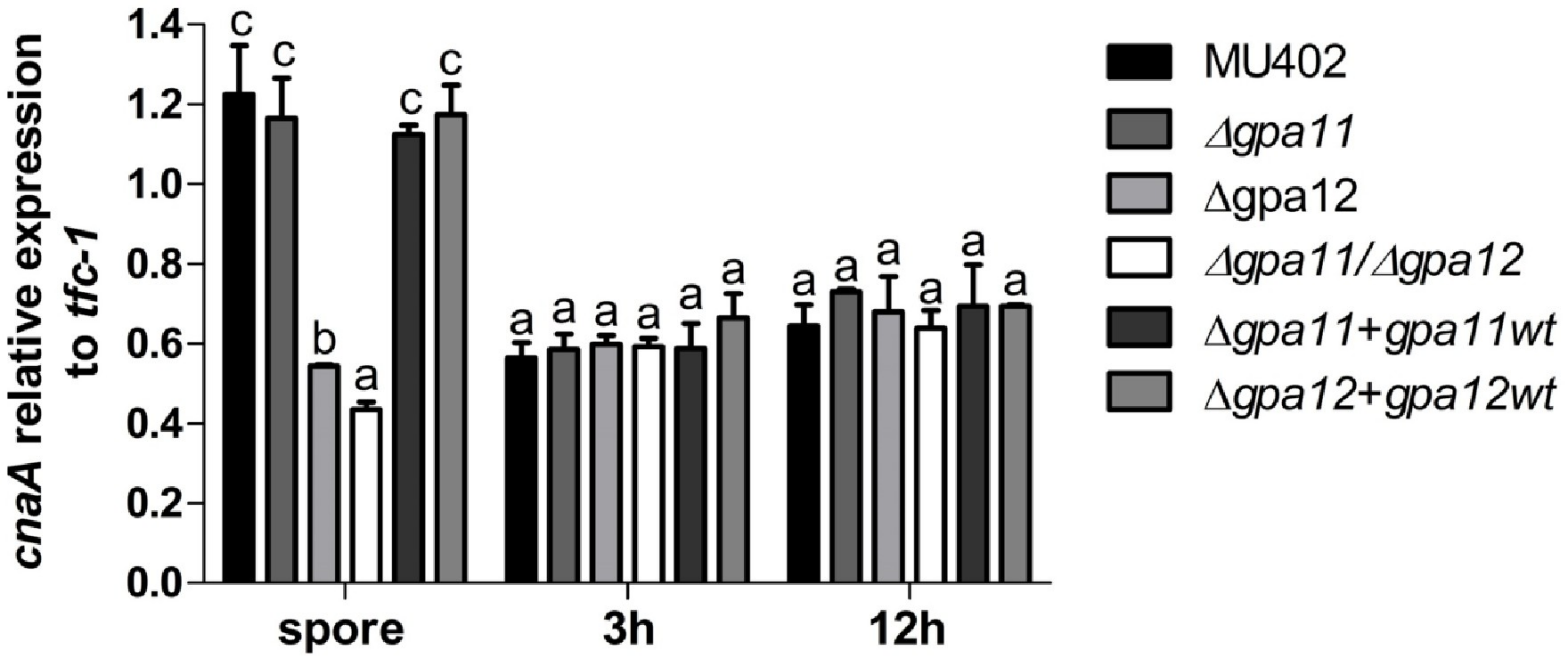
Quantitation of *cnaA* mRNA levels in *M*. *circinelloides* Δ*gpa11* and Δ*gpa12* mutant strains. Expression of *cnaA* relative to *tfc-1* in total RNA purified from sporangiospores and mycelium grown in aerobic conditions after indicated times in YPG. qRT-PCR was performed to determine the transcript levels of *cnaA* and a ΔCt analysis was performed to compare the mRNA level between the strains. Four independent experiments were performed for each condition. Statistically significant differences are indicated by different letters (ANOVA and Fisher’s tests; *p* ≤ 0.05).

The mRNA levels of *cnaA* in the wild-type and Δ*gpa11* mutant spores were similar to each other, while lower mRNA levels were observed in spores from Δ*gpa12* and Δ*gpa11*/Δ*gpa12* mutants by around 56 and 64% respectively, compared to Δ*gpa11*, Δ*gpa11+gpa11*wt, Δ*gpa12+gpa12*wt and wild-type strains (Fig 6). Although the mRNA levels of *cnaA* in mycelium grown for 3 or 12 h in YPG were similar in all strains tested (Fig 6). These results showed that dysfunction of Gpa12 led to down-regulation of *cnaA* mRNA levels in spores of *M*. *circinelloides*. The lower cnaA expression in Δ*gpa11*/Δ*gpa12* suggested that *gpa11* can partially compensate the absence of *gpa12*.

**Fig 7:**
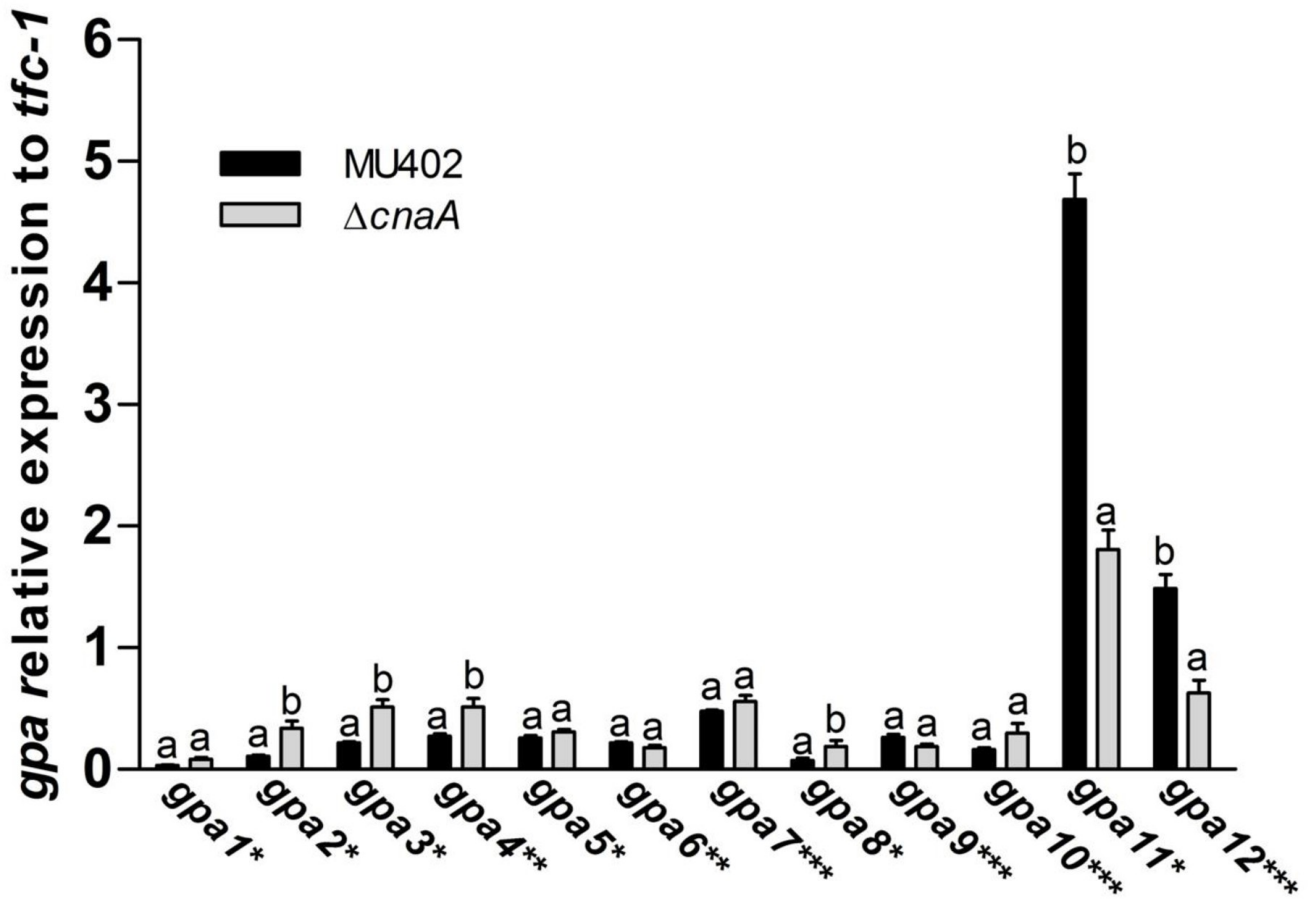
Quantitation of *gpa* mRNA levels in *M*. *circinelloides ΔcnaA*. Changes in levels of mRNA from different *gpa* genes from total RNA purified from sporangiospores of wild-type (closed bars) and Δ*cnaA* (open bars). qRT-PCR was carried out to determine the transcript levels of *gpa* genes, ΔCt analysis was performed to compare the mRNA levels between the strains. Four independent experiments were performed for each condition. Statistically significant differences are indicated by different letters (ANOVA and Fisher’s tests; *p* ≤ 0.05).

To investigate if *cnaA* dysfunction affects *gpa11* and *gpa12* mRNA levels during the spore stage, the transcripts of all *gpa* were quantified by qRT-PCR in spores of the Δ*cnaA* mutant form of *M*. *circinelloides*. The analysis of transcript levels from all *gpa* genes revealed that in the spores from the Δ*cnaA* mutant strain, only *gpa11* and *gpa12* mRNA levels were significantly decreased (61.4 and 57.9%, respectively) compared to the levels from spores of the wild-type (Fig 7). Furthermore, *gpa1*, *gpa2*, *gpa3*, and *gpa8*, whose products belong to group I (Gα_s_), and *gpa4* that belongs to group II showed a significant increase in mRNA levels (1.6–2.3 times) compared to the wild-type (Fig 7, S2 Table). Interestingly, the Δ*gpa11*/Δ*gpa12* mutant strain also showed a significant increase in the mRNA levels of *gpa3*, and *gpa4* (Fig 2). These results suggest a genetic crosstalk between *gpa11*, *gpa12* and *cnaA* gene products.

**Fig 8:**
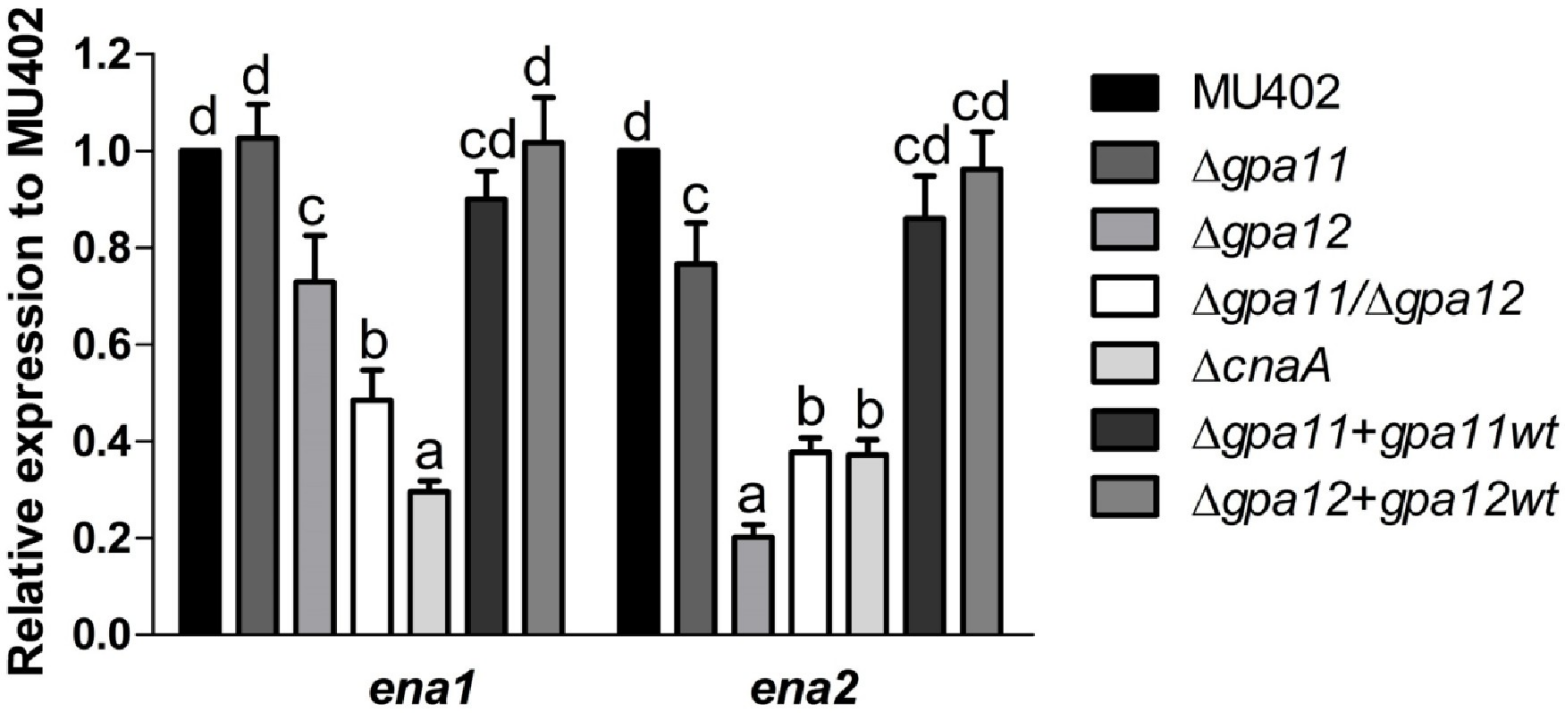
Influence of Gpa11, Gpa12, and CnaA on mRNA levels of *ena* genes in *M*. *circinelloides*. Levels of **A)** *ena1* and **B)** *ena2* measured by qRT-PCR from total mRNA from sporangiospores. ΔΔCt analysis was performed to compare mRNA levels between strains. Four independent experiments were performed for each condition. Different letters denote statistically significant differences (ANOVA, Fisher, p<0.05).

Activation of the calcineurin pathway is downregulated in Δ*gpa11/*Δ*gpa12* since the dysfunction of Gpa11 and Gpa12 led to the down-regulation of *cnaA* mRNA levels in spores, we investigated if elements downstream of the calcineurin pathway could also be deregulated in *gpa*-mutant strains. In the methylotrophic yeast *Hansenula polymorpha*, the transcription of the gene *ena1*, which encodes a plasma membrane Na^+^/K^+^-ATPase, is induced by the activation of the calcineurin signaling pathway [31]. In order to investigate if a similar scenario could be present in *M*. *circinelloides*, we quantified the mRNA levels of *ena* homologues in spores from all strains. Firstly, we identified in *M*. *circinelloides* two Ena homologues, Ena1 and Ena2, with 35.8 and 35.3% identity and 59.2 and 51.6% similarity, respectively, to Ena1 from *H*. *polymorpha* and 63.1 and 77.3% identity and similarity between them (S2 Fig). The mRNA levels of *ena1* and *ena2* from spores of Δ*cnaA* mutant strain were 70.5 and 62.8% lower, respectively, compared to those levels of the wild-type strain (Fig 8). In Fig 8, marked under expression of *ena1* was seen in the double *gpa11*/*gpa12* mutant, while for *ena2*, loss of *gpa12* alone already leads to low expression. For both *ena1* and *ena2*, loss of *gpa11* alone does not have a large effect though it is significant already for *ena2*. In general, this result indicates that *ena1* and *ena2* mRNA levels are under control of CnaA and Gpa12 in *M*. *circinelloides*.

To determine if the calcineurin signaling pathway could be altered in the Δ*gpa11*/Δ*gpa12* mutant strain, a calcineurin inhibitor, FK506 was employed. It had been previously reported that FK506 impaired mycelia growth in the Δ*cnaA* strain [15]. We assessed the capability of spores from the single and double mutant strains in *gpa11* and *gpa12* to develop mycelia growth in the presence of FK506. Our results showed that only the double mutant Δ*gpa11/*Δ*gpa12* strain showed a phenotype similar to that of the Δ*cnaA* strain when grown on a poor nitrogen source (YNB) supplemented with FK506, while *gpa*-single mutant strains showed a phenotype similar to the wild-type strain independent of the nitrogen source (S3A Fig). It has been shown that the *cnaA* mutation also leads to increased sensitivity to detergent in a rich YPD media [15]. Our results showed that Δ*gpa11* and Δ*gpa12* or double *gpa*-mutant strains were also more sensitive to the detergent Triton X-100 than the wild type strain in YPG or YNB media (S3B Fig).

### The role of *gpa11* and *gpa12* in the virulence of *M*. *circinelloides*

**Fig 9:**
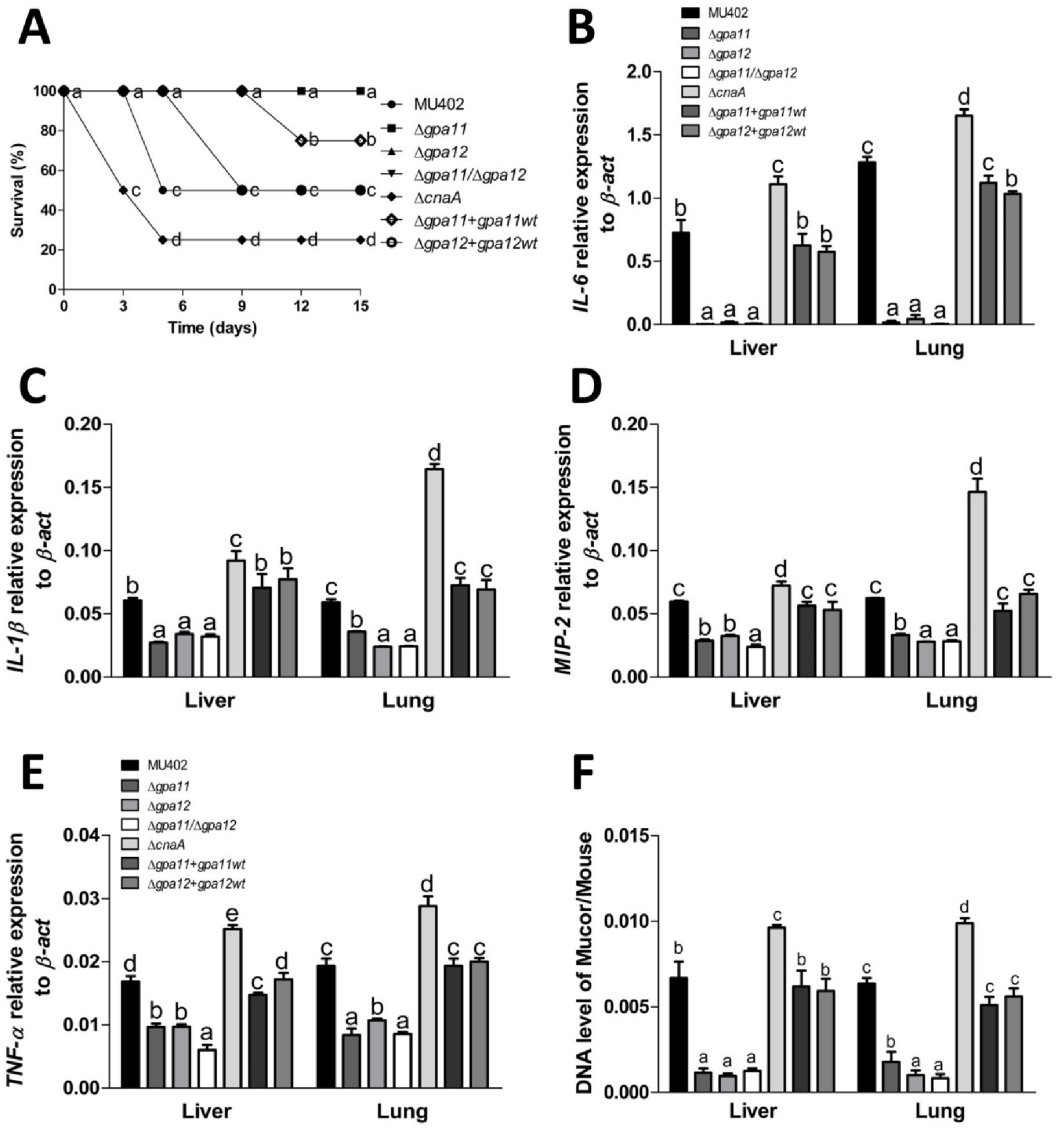
Role of *gpa11* and *gpa12* in the virulence of *M*. *circinelloides*. **A)** Survival of diabetic mice infected intraperitoneally with 2 × 10^7^ sporangiospores from different *M*. *circinelloides* strains recorded each day post-injection. N = 8, two independent experiments. Relative expression of inflammation markers **B)** *IL-6*, **C)** *IL-1β*, **D)** *MIP-2*, **E)** *TNF-α* used to monitor tissue damage in the liver and lung of mice infected with spores from the different *M*. *circinelloides* strains (N = 4). **F)** Fungal burden, determined by qPCR, from the liver and lung of infected mice. Three independent experiments were performed for each condition. Statistically significant differences are indicated by different letters (ANOVA and Fisher’s tests; *p* ≤ 0.05).

The increase in spore size and faster germination rate are associated with virulence increase in *M*. *circinelloides* [17]. Based on the fact that the spores of Δ*gpa11*/Δ*gpa12* mutant strain are much larger, germinate faster, and cause a decrease in mRNA levels of *cnaA* compared to the wild-type strain, we performed virulence assays. In the mouse killing assay, diabetic mice were used which simulates one of the risk factors for acquiring mucormycosis [18]. Mice were injected intraperitoneally with 2 × 10^7^ spores/mouse from each strain and mouse survival was monitored each day. Reduced survival was observed only in the Δ*gpa11+gpa11*wt, Δ*gpa12+gpa12*wt, Δ*cnaA* and wild-type strains. 50 and 75% of the mouse population died after 5 days post-inoculation with the spores from wild-type and Δ*cnaA* strains, respectively (Fig 9A). We quantified by qRT-PCR the mRNA levels of the inflammation markers *IL-6*, *IL-1β*, *MIP-2* and *TNF-α* in the liver and lung of mice infected with the different strains (Fig 9B–9E). The mice infected with the Δ*gpa11+gpa11*wt, Δ*gpa12+gpa12*wt and wild-type strains, showed significantly increased mRNA levels of *Il-6* (130 times in liver and 124 times in lung), *Il-1β* (96% in liver and 118% in lung), *MIP-2* (115% in liver and 110% in lung) and *TNF-α* (109% in liver and 112% in lung) compared to mRNA levels from animals infected with *gpa*-mutants (Fig 9B–9E). Also, the Δ*cnaA* mutant strain produced significantly higher mRNA levels of all inflammation markers in liver (21–53%) or lung (28–178%) compared to the wild-type strain (Fig 9B–9E). These results indicate that mice infected with the Δ*gpa11*, Δ*gpa12*, or the Δ*gpa11*/Δ*gpa12* mutants induce a weaker inflammatory response compared to those infected with the Δ*cnaA*, Δ*gpa11+gpa11*wt and Δ*gpa12+gpa12*wt or wild-type strains.

Fungal burden was quantified in tissues (liver and lung) of the mice infected with all the strains by qPCR using the *tfc-1* as gene marker for *M*. *circinelloides* [29]. The quantity of *tfc-1* from the Δ*gpa11*, Δ*gpa12*, the Δ*gpa11*/Δ*gpa12* mutant strains were lower (up to 82% in liver and 87% in lung) than in those infected with the wild-type strain. Moreover, the Δ*cnaA* strain fungal burden was 26 and 32% more in liver and lung, respectively, compared to the wild-type strain (Fig 9F). These results indicate that mice infected with spores from Δ*gpa11*, Δ*gpa12*, or the Δ*gpa11*/Δ*gpa12* mutant strains induce a weaker inflammatory response and less mouse tissue invasion compared to those infected with the Δ*cnaA*, complemented or wild-type strains.

**Fig 10:**
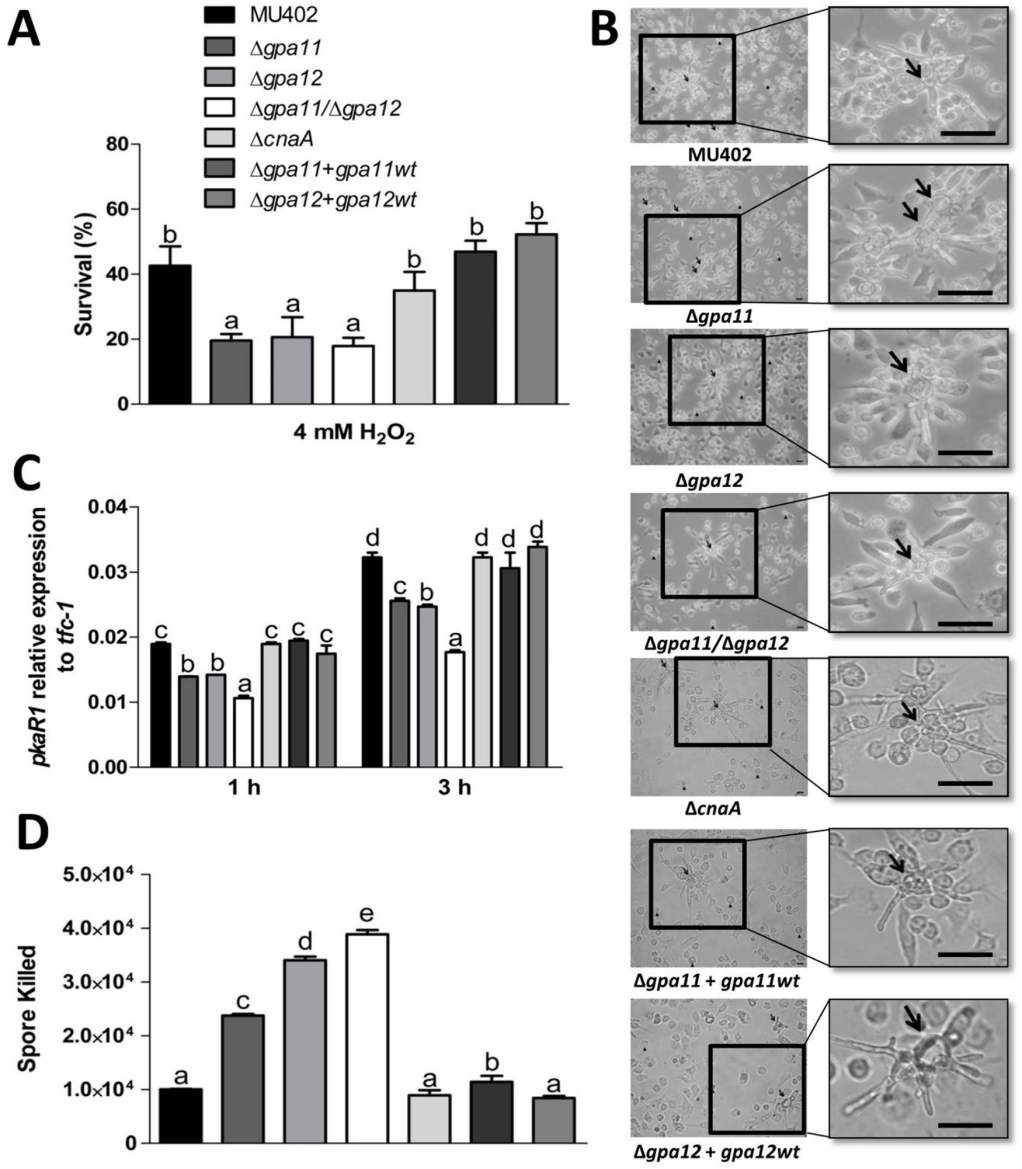
Effect of mutations in *gpa11* and *gpa12* in the viability of sporangiospores from *M*. *circinelloides* in H_2_O_2_ and macrophage interaction. **A)** Spores from different strains were treated or not with 4 mM H_2_O_2_ and incubated at 28 °C on YPG. Survival was obtained from colonies grown for 24 h from the quotient of colonies from treatment versus no treatment. **B)** Germination of spores during macrophage interaction after 3 h was observed under direct observation by light microscopy (40 X) Scale bar = 20 μm. Arrows indicate germinating hyphae and head of arrows indicate swelling spores. **C)** The *pkaR1* mRNA levels were quantified by qRT-PCR after 1 and 3 h of spore and macrophage interaction. **D)** Quantitation of killed spores by qPCR using *tfc-1* after 3 h of incubation with mouse macrophages. A ΔCt analysis was performed to compare the mRNA and gene levels between the samples. Figures show the average of three independent experiments. Statistically significant differences are indicated by different letters (ANOVA and Fisher’s tests; *p* ≤ 0.05).

To explain the mechanism of low virulence of the Δ*gpa11/*Δ*gpa12* spores despite their bigger size, we tested the effect of H_2_O_2_ on spore survival as previously reported [32, 33]. The Δ*gpa11*, Δ*gpa12*, and the Δ*gpa11*/Δ*gpa12* mutant strains showed a significant decrease in spore survival compared to the wild-type strain in YPG (up to 55%) media supplemented with H_2_O_2_. The lowest levels of survival were registered for Δ*gpa11/*Δ*gpa12* spores, compared to the wild-type. While Δ*cnaA* strain showed no significant difference in survival rate compared to the wild-type strain (Fig 10A), meanwhile the complemented mutant strains showed similar survival rate as the wild-type strain after the challenged with H_2_O_2_.

Based on this result, we conducted a confrontation assay with all the *M*. *circinelloides* strains against the murine macrophage cell line RAW264.7. After 3 h of interaction of macrophages and spores, we observed spore germination in presence of the macrophages (Fig 10B). The germination rate was quantified indirectly by the mRNA levels of *pkaR1* by qRT-PCR, which is used as a molecular marker for hyphae development [28], after 1 or 3 h of macrophage and spore interaction. We observed lower *pkaR1* transcript levels after 1 (≈26%) and 3 h (≈22%) in the single *gpa*-mutant strains compared to the wild-type strain (Fig 10C). The Δ*gpa11/*Δ*gpa12* mutant strain showed the lowest levels of *pkaR1* mRNA after 1 (≈44%) or 3 h (≈45%) compared to the wild-type (Fig 10C), meanwhile the complemented mutant strains showed similar survival rate as the wild-type strain after the challenged with H_2_O_2_. Based on a standard curve using different amounts of spores from *M*. *circinelloides*, we quantified by qPCR the *tfc-1* levels from spores that were digested by macrophages after the interaction as described previously [32, 33]. The spores from the Δ*gpa11+gpa11*wt, Δ*gpa12+gpa12*wt, Δ*cnaA* and wild-type strains showed fewer spores killed after the interaction with the macrophages (Fig 10D) compared to the Δ*gpa11*, Δ*gpa12* and Δ*gpa11/*Δ*gpa12* mutant strains that showed more spores killed in this assay (Fig 10D). These results indicate that the spores from the Δ*gpa11/*Δ*gpa12* mutant strain are the most susceptible to the macrophages compared to the spores from the Δ*cnaA*, Δ*gpa11+gpa11*wt, Δ*gpa12+gpa12*wt and wild-type strains. This could be explained, in part, by their higher susceptibility to oxidative damage.

## Discussion

*M*. *circinelloides* produces several types of spores through its life cycle, and each type of spore has a specific cellular fate [2, 3]. The most well described spore is the sporangiospore, which is an asexual spore and is produced after the mycelium reaches a certain stage on solid surfaces, producing an aerial structure known as the sporangiophore. Several factors have been described that contribute to sporangiospore production in *M*. *circinelloides*; for example, light is a positive stimulator of sporulation and carotenogenesis [34]. The detailed molecular mechanism of how sporangiospores are produced in *M*. *circinelloides* is still not known. Transcript levels from *gpa11* and *gpa12* from *M*. *circinelloides*, have been shown to mainly accumulate in the sporangiospore, suggesting an important function either in spore formation, production, and/or germination [25].

**Fig 11:**
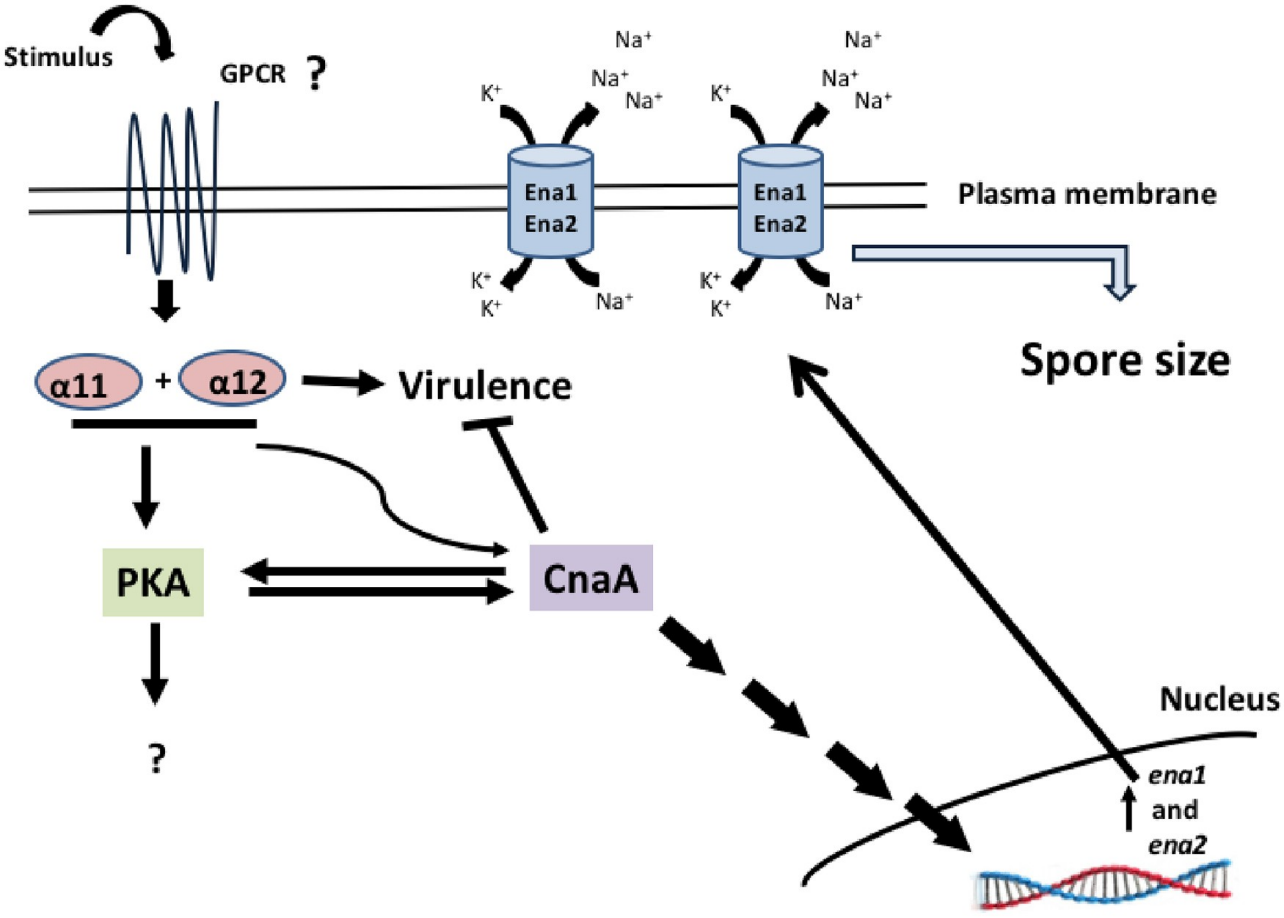
Proposed model of the function of Gpa11 and Gpa12 regulating sporangiospore size and virulence in *M*. *circinelloides*. Through one or more GPCRs, Gpa11 and Gpa12 in the spores of *M*. *circinelloides* activate CnaA, which in turn activates the calcineurin pathway. The calcineurin pathway leads to the expression of genes regulated by this pathway, like *ena1* and *ena2*. The dysfunction of Ena1 and Ena2, which are Na^+^-K^+^ ATPases, could increase cytosolic levels of sodium ions, allowing the influx of water, leading to an increase in spore size in *M*. *circinelloides* as was observed in the Δ*gpa11*/Δ*gpa12* strain. The CnaA negatively regulates the virulence and stimulates the PKA pathway, meanwhile Gpa11 or Gpa12 positively regulate the virulence and activate the PKA pathway during aerobic germination, but unknown elements are implied in the avirulent phenotype by the mutations in *gpa11* and *gpa12*.

Our work showed that *gpa11* and *gpa12* has an important function in the spore since Δ*gpa12* and specially Δ*gpa11*/Δ*gpa12* showed increased spore sizes compared to Δ*gpa11* and wild-type strains. Spore size in *M*. *circinelloides* is known to be controlled by the calcineurin pathway. Thus, mutation of *cnaA*, which encodes the catalytic subunit A of calcineurin, leads to large spore size, faster germination, and increased virulence, among other defects [15]. Interestingly, *cnaA* mRNA levels in spores of Δ*gpa12* and Δ*gpa11/*Δ*gpa12* strains were lower than those of the wild-type strain, suggesting that *gpa12* positively controls the levels of *cnaA* in the spore, a function that can be compensated by *gpa11* in absence of *gpa12* as indicated the more severe effect observed in Δ*gpa11/*Δ*gpa12* compared to Δ*gpa12*. Moreover, the mRNA levels of *ena1* and *ena2*, used as a marker for the activated calcineurin pathway [31], were decreased in Δ*gpa12*, Δ*gpa11/*Δ*gpa12* and Δ*cnaA* strains compared to the wild-type strain. Additionally, the spore size increase could be result of the low *ena1* and *ena2* expression because dysfunction of Ena homologues leads to cell swelling in human renal cells, as dysfunction of Na^+^-K^+^ ATPases leads to the accumulation of sodium ions in cells, which allows water entry into the cell to compensate the osmotic gradient [35]. Also, *Saccharomyces cerevisiae* mutants in the regulatory subunit of the calcineurin decreased the expression of ENA1 and accumulated Na^+^ and Li^+^ ions [36]. Therefore, it is tempting to speculate that something similar occurs in the Δ*gpa11*/Δ*gpa12* strain of *M*. *circinelloides*, it will be interesting to know if the mutation of *ena1* or/and *ena2* could altered the spore size in this Mucoral (Fig 11). Overall, these findings suggest that the calcineurin pathway is under the control of Gpa12, and Gpa11 in a lesser extent, in spores from *M*. *circinelloides*, and consequently absence of *gpa12* and *gpa11* or *cnaA* results similar phenotypes such as spore enlargement (Fig 4), accelerated germination (Fig 5) and sensibility to the calcineurin inhibitor FK506 (S2 Fig). In addition, the double mutation of *gpa11* and *gpa12* led to lower cAMP levels and PKA activity compared to the the Δ*gpa11* or Δ*gpa12* after 3 hours of aerobic growth (Fig 5). Similar levels of cAMP and PKA activity between Δ*gpa11/*Δ*gpa12* and Δ*cnaA* were observed (data not shown), indicating a crosstalk in the regulation of PKA pathway between Gpa11/Gpa12 and CnaA (Fig 11). Interestingly, although Δ*gpa12* decreased the mRNA from *cnaA*, this mutation did not alter the levels of cAMP and PKA activity, suggesting different pathways in the regulation of activation of PKA by Gpa12 or Gpa11/Gpa12.

Additionally to the regulation of the calcineurin pathway by *gpa12* and *gpa11*, the calcineurin pathway also regulates the expression of *gpa12* and *gpa11* in the spore since their mRNA levels was down regulated in the Δ*cnaA* strain (Fig 7). This observation revealed a regulatory crosstalk between *gpa12* and *gpa11* and the calcineurin pathway. Alteration of this regulatory system by deletion of some key element seems to affect the expression of other *gpa* genes. Thus, Δ*cnaA* strain showed a significant mRNA increase of *gpa2*-*4*, which codes for products belonging to Group I (Gα_s_) and Group II of the Gα phylogeny. The mRNA levels of *gpa3* and *gpa4* were also increased in the Δ*gpa12* and Δ*gpa11*/Δ*gpa12* mutant strains, suggesting a correlation between *gpa11* with *gpa12* and *cnaA* in terms of regulation of *gpa3* and *gpa4* mRNA levels in spores from *M*. *circinelloides*, although more experiments are needed to clarify this possible correlation.

Deletion of *M*. *circinelloides* in *cnaA* showed an elevated virulence (Fig 9) that has been attributed in part to the larger spore size that result in increased germination rate, allowing faster destruction of the host [17]. Surprisingly, single and double mutants of *gpa11* and *gpa12* were completely avirulent in a diabetic mouse model, despite the Δ*gpa11*/Δ*gpa12* displayed low *cnaA* mRNA levels and mutant produce large spores that show high germination rate compared to the wild-type strain (Fig 5). Moreover, the inability to infect mice could not be attributed to a poor growth of the *gpa11* and *gpa12* mutants (Fig 3). The defect in virulence of *gpa11* and *gpa12* mutants and the higher virulence of the mutant of *cnaA* were confirmed by the figures of fungal burden and the levels of chemokines (*IL-6*, *IL-1β*, *TNF-α*, and *MIP-2*) known to be induced under filamentous infections [37–39], including *M*. *circinelloides* [40].

The single and double mutants of *gpa11* and *gpa12* did not show a significant increase in the mRNA levels of these inflammation markers in diabetic mice tissues compared to the levels from the wild-type or Δ*cnaA* strains, despite larger spore size and higher germination rate of Δ*gpa11*/Δ*gpa12* mutant compared to the wild-type strain. Despite these similarities, spores of Δ*gpa11*/Δ*gpa12* and Δ*cnaA* strains behave in a totally different manner in terms of H_2_O_2_ resistance, since Δ*cnaA* spores are more resistant to H_2_O_2_ than those from Δ*gpa11*/Δ*gpa12*, and also single mutants of this genes (Fig 10). Moreover, it has been also reported that the spores from Δ*cnaA* strain are more resistant to phagocytosis [15], but the single and double *gpa*-mutant strains were more efficiently killed by macrophages than spores from the wild-type strain. This result could in part explain that *gpa11* and *gpa12* mutants are unable to infect diabetic mice (Fig 9) The combination of large spores and inability to cause infection shown by mutants of *gpa11* and *gpa12*, especially the double mutant, suggests that both phenotypes are controlled by the same regulatory pathway (Fig 11). This regulatory pathway in some point diverge in two branches, one that controls spore size through the calcineurin pathway and another that regulates the expression of genes important for the virulence. This model could explain the higher virulence of *cnaA* mutants that keep functioning the virulence-specific regulatory branch and simultaneously produce large spores with accelerated germination, it seems that a total lack of CnaA is needed to display a virulent phenotype. Therefore, *gpa11* and *gpa12* define a signal transduction pathway that play pivotal role in fungal infection in *M*. *circinelloides*. The dissection of this pathway with the identification of receptor to unknown signals, other components of the signal cascade, and targets genes will provide a better knowledge of the understudied process of fungal colonization by Mucorales.

Our work is important to the fungi field revealing new hypothesis to test. Fungal spore size has been found to be an important virulence determinant in fungal pathogens including ascomycetes (*Beauveria bassiana*, *Botrytis cinerea*), basiodiomycetes (*Cryptococcus neoformans*) or mucorales (*Mucor circinelloides*). In the entomopathogenic fungus *B*. *bassiana*, when the vacuolar ATPase subunit H encoding gene was disrupted, conidia diameter was reduced by 16% when compared to the wild-type strain. Moreover, this mutant displayed significantly attenuated virulence against the *Galleria mellonella* larvae [41]. In both fungal phytopathogens *B*. *cinera* and *M*. *fructicola*, environmental conditions regulate spore size. It has been reported that in *B*. *cinerea*, when grown on culture medium that contains 30% glucose (w/v), spores were about 50% larger than those grown on 2% glucose (w/v). Notably, these larger spores had enhanced aggressiveness against the flowers of *Rosa hybrida* [42]. Comparative analysis of spore sizes from different *C*. *neoformans* strains revealed that spores from serotype A are around 10% larger in diameter when compared to the serotype D [43]. Noteworthy, serotype A is the major responsible of *C*. *neoformans* infections (95%) and it has been associated with more severe infections in immunocompromised patients [44]. Interestingly, to the best of our knowledge, the first report for larger fungal spores (>10 μm in diameter) In *M*. *circinelloides* that does not imply a more virulent phenotype when compared to the wild-type strain.

Our work provides evidence for a genetic and functional linkage between *gpa11* and *gpa12* with the calcineurin pathway in *M*. *circinelloides*, suggesting that *cnaA* is under control of *gpa11* and *gpa12* and these elements are associated with a larger spore phenotype. However, larger spore phenotype by itself is not a prerequisite for virulence, suggesting that *gpa11* and *gpa12* also regulate a set of genes that are specifically involved in virulence.

## Methods

### Fungal strains, culture media, and growth conditions

*M*. *circinelloides* f. *lusitanicus* MU402 (*leuA*^-^, *pyrG*^-^) [27] was used for the *gpa11* and *gpa12* gene deletion studies. Additionally, the MU636 strain (*leuA*^-^, *pyrG*^+^) derived from *M*. *circinelloides* MU402 was also employed in this work [45], although it is not shown, this strain exhibits similar phenotypes as the wild-type strain MU402. A *M*. *circinelloides* Δ*cnaA* mutant strain [15] derived from MU402 was kindly provided by Dr. Soo Chan Lee (University of Texas at San Antonio, USA). Yeast–peptone–glucose (YPG) medium (pH 4.5); 1 L medium containing 3 g yeast extract (BD Bioxon, USA), 10 g gelatin peptone (BD Bioxon, USA), 20 g glucose (BD Bioxon, USA). Yeast nitrogen base (YNB) (Sigma, USA) or MMC (minimal medium with casamino acids), which contained 10 g/L casamino acids (BD Difco, USA), 20 g/L glucose, and 0.5 g/L yeast nitrogen base, supplemented with uracil (200 mg/L) (Sigma, USA) as required; 15 g/L agar (BD Bioxon, USA) was added when solid media were prepared. For spore production and culture conditions, germination under self-anaerobic and aerobic atmosphere, as well as the quantitation of growth, spore size, and hyphae length protocols described previously were followed with no modifications [18, 46].

100 spores from the different strains were treated or not with 4 mM H_2_O_2_ for 1 h at 4 °C and spread on YNB or YPG plates and incubated for 24 h at 28 °C. YNB or YPG solid media were supplemented or not with 0.005% Triton X-100 and 100 spores from different strains were spread on the plates, incubation was carried out for 1 day at 28 °C.

### Targeted deletion in *M*. *circinelloides*

#### Targeted deletion of *gpa11*

Using genomic DNA from MU402 wild-type strain *of M*. *circinelloides* 1.1-kb fragments that correspond to the 5′ upstream or 3′ downstream regions from the start or stop translation codon from *gpa11*, respectively, were amplified by PCR. The pair of oligonucleotides Gpa11-P-Fwd and Gpa11-P-Sac-Rev was used to amplify the 5′ region, with a *Sac*l site added to the 3′ end of the Gpa11-P-Sac-Rev primer. The oligonucleotides Gpa11-T-Sac-Fwd and Gpa11-T-Xba-Rev were used to amplify the 3′ region, with a *Sac*I site added to the 5′ end of the Gpa11-T-Sac-Fwd primer and an *Xba*l site to the 3′ end of the Gpa11-T-Xba-Rev primer. A 2-kb fragment that corresponds to the *pyrG* gene was PCR-amplified using the primers PyrG-Sac-Fwd and PyrG-Sac-Rev and *Sac*I sites were added to its 5′ end and 3′ end (S3 Table).

The 5′ and 3′ fragments from *gpa11* and *pyrG* were independently cloned into pJET1.2/blunt resulting in the pGpa11-5′ (for the *gpa11* 5´-region), pGpa11-3′ (for the *gpa11* 3′-region), and pPyrG (for the *pyrG* gene) vectors. The *Sac*l sites in the different fragments allowed the subcloning of *pyrG* into pGpa11-5′, resulting in pGpa115′-pyrG. Finally, 1.1-kb from the 3′ region of *gpa11* was cut using the *Sac*l and *Xba*l enzymes, and subcloned into pGpa115′-pyrG—the resulting plasmid was named pGpa115′-pyrG-3′. This resulting plasmid was used as template to obtain the recombinant fragment (Gpa115′-pyrG-3′ of 4.2 kb) by PCR with the oligonucleotides Gpa11-P-Fwd and Gpa11-T-Xba-Rev. All recombinant plasmids were confirmed by PCR and restriction enzyme patterns.

#### Targeted deletion of *gpa12*

The *gpa12* recombinant fragment coupled with the *pyrG* selection gene was generated through overlapping-PCR. In this process, three PCR fragments containing overlapping sequences between them were PCR-amplified using the following oligonucleotides (S3 Table): 12pUFow-Pstl and 12pURev-pyrG, which amplified 1-kb from the 5′ upstream region of the start translation codon of *gpa12*; and oligonucleotides 12pDRev-Notl and 12pDFow-pyrG, which amplified 1-kb from the 3′ downstream region of the stop translation codon of *gpa12*. In oligonucleotide 12pURev-pyrG, an added nucleotide sequence at the 3' end hybridized to the 5′ end of *pyrG*; and in oligonucleotide 12pDFow-pyrG, an added nucleotide sequence at its 5' end hybridized to the 3' end of *pyrG*. The gene *pyrG* was PCR-amplified using the oligonucleotides PyrG-Sac-Fwd and PyrG-Sac-Rev, with pMAT1700 [47] as a template. Three individual PCR reactions were performed to obtain 3 DNA fragments. The *gpa12* 5′ region, *pyrG* (2 kb), and the *gpa12* 3′ region, which were all purified. Overlapping-PCR was performed using 100 ng/μL of each template; 10 μM each of the oligonucleotides 12pUFow-Pstl and 12pDRev-Notl and 1 μL of Herculase II Fusion Enzyme (Agilent Technologies, USA), at a molar ratio of 1:1:1, were used to generate the recombinant fragment of *gpa12*.

Targeted deletion of *gpa11* in Δ*gpa12* strain: To generate the Δ*gpa11*/Δ*gpa12* double-mutant strain, the plasmid pGpa115′-pyrG-3’-previously obtained for the *gpa11* mutation was used. The gene *leuA*, which is required for leucine synthesis [48], was obtained by PCR with the oligonucleotides leuA-Sac-Fwd and leuA-Sac-Rev, and *Sac*I restriction sites were added to both oligonucleotides at their 5′ ends (S3 Table), and then annealed into the plasmid pLeu4 [49]. Both *leuA* (3.7 kb) and the pGpa113′-pyrG-5′ vector were digested with *Sac*I, and both products were ligated using the enzyme T4 DNA ligase (Promega, USA); the resulting plasmid was named pGpa115′-leuA-3′. The plasmid pGpa115′-leuA-3′ was used as a template and using the oligonucleotides Gpa11-P-Fwd and Gpa11-T-Xba-Rev, PCR amplification was carried out to obtain the recombinant fragment (5.9-kb) to delete *gpa11* in the Δ*gpa12* strain. All recombinant plasmids were identified by PCR and restriction fragment patterns.

Protoplasts obtained from *M*. *circinelloides* MU402 were transformed with the recombinant DNA fragments (3 μg/transformation) according to a previously described protocol [50]. Transformants were obtained under selective conditions on MMC agar plates lacking uracil for single deletion mutants or YNB agar plates lacking uracil and leucine for the double mutants. Monosporic cultures were obtained after 5 cycles of sporulation in either selective medium or rich medium, at which point, 100% of the spores could grow on the selective medium, indicating the transformation of all nuclei.

### Molecular identification of *gpa11* and *gpa12* mutant strains from *M*. *circinelloides*

#### PCR confirmation of *gpa11* gene deletion

Transformed strains were identified by PCR using the oligonucleotides 11-C-Fwd (forward) and pyrG-R2 (reverse) (S3 Table); 11-C-Fwd hybridized outside the recombination fragment used to delete the *gpa11* gene and pyrG-R2 (S3 Table) that hybridize in the selectable marker sequence to determine a homologous integration event, a positive recombination event was determined by PCR amplification of 1.4-kb band. A lack of amplification band indicated the wild-type genotype (S4 Fig).

#### PCR confirmation of *gpa12* gene deletion

Using a similar approach, the transformants were identified by PCR with the pair of oligonucleotides 12-C-Fwd, which hybridizes outside the recombination fragment used to delete *gpa12* gene, and pyrG-R2 (S3 Table), which hybridizes in the selectable marker sequence. Those which generated an amplicon of 1.3-kb was the mutant genotype, but non-amplification confirmed a wild-type genotype. (S4 Fig).

#### PCR confirmation of double mutant Δ*gpa11/*Δ*gpa12*

Using the oligonucleotides 11-C-Fwd (forward) and leuA-C-Rev (reverse) (S3 Table); 11-C-Fwd hybridized outside the recombination fragment used to delete the *gpa11* gene and leuA-C-Rev (S3 Table) hybridized in the selectable marker sequence to determine a homologous integration event. A positive recombination event was determined by PCR amplification of 2.2-kb band, while no amplification confirmed the wild-type genotype (S4 Fig).

As a positive control for DNA integrity that was used for PCR reactions, the pair of oligonucleotides, Gpa11-T-Sac-Fwd / Gpa11-T-Xba-Rev, which hybridized with the 3′-end region of *gpa11* were used for Δ*gpa11* and a 1.1-kb amplicon indicated a positive reaction. For Δ*gpa12* the pair of primers, 12pUFow-Pstl / 12pURev-pyrG, which hybridized with the 5’ region of *gpa12* were used and 1.1 kb was considered as positive control. The pair of oligonucleotides 11-C-Fwd / Gpa11-P-Sac-Rev was used in a PCR reaction for positive controls for Δ*gpa11*/Δ*gpa12* and a 1.3 kb amplicon indicated a positive PCR reaction; the wild-type strain also amplified an identical PCR size band as well as each mutant strain (S4 Fig).

### Southern blot to identify the deletions of *gpa11* and *gpa12*

A total of 1 μg of genomic DNA from MU402 and the mutants of *M*. *circinelloides* were digested with indicated restriction enzyme. DNA was electrophorized and transferred to nylon membranes (HybondTM-N+, Amersham Biosciences, UK) following the recommended protocol [51]. Southern blot hybridization was performed under stringent conditions. DNA probes were labelled with [α-32P] dCTP using Ready-To-Go Labeling Beads (GE Healthcare Life Science). For Southern blot experiments, a DNA probe (1 kb) was directly amplified from genomic DNA from *M*. *circinelloides* using the primer pairs that amplified the 5′ region upstream of the start translation codon from *gpa11*, for *gpa12* identification a fragment (1 kb) corresponding to the 3′ region downstream from the stop translation codon was used as a probe and finally the primer pairs that amplified the 3′ region downstream from the stop translation codon (1 kb) from *gpa11* for the double mutation.

### Complementation of Δ*gpa11* and Δ*gpa12* strains

The *gpa11* and *gpa12* wild-type ORFs were PCR-amplified using the genomic DNA of *M*. *circinelloides* MU402 as the template and the oligonucleotides *gpa11*-FWR-*Xho*I/*gpa11*-REV-*Not*I and *gpa12*-FWR-*Sal*I/*gpa11*-REV-*Not*I (S3 Table), which introduced restriction sites for *Xho*I and *Not*I at the 5' and 3' ends, respectively. Wild-type *gpa11* and *gpa12* genes were cloned in pEUKA4 vector [28] under the control of *gpd1* promoter from *M*. *circinelloides* in the *Xho*I and *Not*I sites; the resulting plasmids were confirmed based on restriction patterns and DNA sequencing. Both recombinant plasmids were used to transform the Δ*gpa11* and Δ*gpa12* strains and selection was performed in YNB media (Difco, Franklin Lakes, NJ, USA) medium.

### qRT-PCR confirmation

Total RNA isolated from *gpa11* and *gpa12* single and double mutant strains was used as a template and qRT-PCR was performed to determine all *gpa* transcript levels, as described previously [25].

### Specimen preparation for scanning electron microscope (SEM) imaging of spores

SEM was performed according to Oshell 1997 [52], 4 h post-fixation with osmium tetroxide (1% diluted in cacodylate buffer 0.01 M, pH 7.4). The samples were dehydrated in ethanol solutions of 60, 70, 80 and 90%, and three times in 100% ethanol for 15 min each.

For hexamethyldisilazane (HMDS) drying the samples were rinsed in ethanol- HMDS in proportions of 2:1, 1:1, 1:2, 1:3, and HMDS 100% for 15 min each. The samples were transferred to a desiccator for 24 h. Samples were mounted on stubs and examined with a Zeiss GeminiSEM scanning electron microscope.

### Ethics statement

The ethics agreement was made for the mouse virulence model protocol following the recommendations of the Mexican Federal Regulations for the Use and Care of Laboratory Animals (NOM-062-ZOO-1999) (Especificaciones técnicas para la producción, cuidado y uso de los animales de laboratorio/Technical specifications for production, use and care of laboratory animals) [53]. The Internal Biosecurity and Bioethics Committee of Instituto de Investigaciones Químico Biológicas de la Universidad Michoacana de San Nicolás de Hidalgo firstly reviewed the experimental protocols and authorized them (trade number 06-13/2016).

### Mouse virulence assays

To assess the virulence of *M*. *circinelloides* strains, we used methods described previously [18, 54] with some modifications. Briefly, each group consisting of eight male BALB/c mice (12–16 weeks old, weighing ~20 g, obtained from CINVESTAV, Zacatenco. México) were treated with streptozotocin (200 mg/kg) (Sigma, USA) to induce a diabetic state (>300 mg/dL glucose in blood), and then independent mice groups were inoculated with 2×10^7^ spores from each strain of *M*. *circinelloides*. The spores were suspended in saline solution and injected intraperitoneally into mice, and survival of mice was monitored daily. Three independent assays were conducted for each group.

### Macrophages spore killing assay

Mouse monocyte/macrophage RAW 267.4 (TIB-71) was purchased from American Type Culture Collection (ATCC). Cells were maintained in DMEM medium supplemented with 10% FBS (Sigma, USA), 100 U/mL penicillin (Sigma, USA), and 100 μg/mL streptomycin (Sigma, USA) (basal medium) at 5% CO_2_ and 37 °C for 24 h before treatments.

Macrophages were seeded at 8 × 10^5^ cells/well in DMEM basal medium (GIBCO, Thermo Fisher Scientific, USA) without antibiotics and incubated at 37 °C in 6-well plates. After incubation overnight, the basal medium in each well was replaced with fresh medium, then the macrophages were co-cultured with 2 × 10^5^ spores produced in YPG for 1 or 3 h. Immediately, the supernatants and cells and spores were removed and placed in independent tubes. Then, cells and spores were centrifuged at 1,500 x g for 5 min. Pellet was recovered and frozen until nucleic acid extraction was performed.

### Total *M*. *circinelloides* RNA and DNA isolation from *in vitro* cultures, from macrophages or from mouse tissues

Total RNA and genomic DNA from *M*. *circinelloides*, mice tissues and macrophages were isolated using RNAeasy mini kit and QIAamp DNA Mini Kit, respectively (Qiagen, Venlo, Netherlands).

*M*. *circinelloides* cultures were obtained by filtration in 5 μm membrane filters (Millipore, USA), the mouse tissues were collected following infection with *M*. *circinelloides* after 15 days of spore inoculation. Approximately 25–50 mg of tissue sample from each mouse was transferred into a tube with MagNA Lyser Green Beads (Roche, Switzerland) pre-cooled on ice. Denaturing RLT buffer (700 μL) from the RNeasy Mini Kit or buffer ATL (180 μL) of QIAamp DNA Mini Kit were added immediately before homogenization to obtain total RNA or genomic DNA, respectively. For cell disruption, tubes were placed in the MagNA Lyser Instrument (Roche, Switzerland) and processed twice at 5,500 x g for 40 s with cooling on ice for 1 min between each step. Then, samples were centrifuged for 1 min at 20,000 *x g* (Eppendorf 5417) and the supernatants were used for total RNA or genomic DNA isolation using the corresponding kit, according to the manufacturer’s instructions. To eliminate DNA contamination from RNA samples, samples were treated with DNase I (Promega, USA) according to the manufacturer’s protocol. The RNA was eliminated from DNA samples using RNAse A (Roche, Switzerland). RNA or genomic samples were separated on non-denaturing 2% and 1% agarose gel, respectively stained with ethidium bromide (Sigma, USA), visualized using a Gel Doc XR+ Imager (Bio-Rad, Hercules, CA, USA), and quantified using a SmartSpec Plus spectrophotometer (Bio-Rad).

### Determination of abundance of DNA from *M*. *circinelloides* by qPCR

Abundance of *M*. *circinelloides* from mice tissues or macrophages was compared based in a spore standard curve of *M*. *circinelloides*. MU402 spore concentrations between 1 x 10^2^ and 1 x 10^7^ mL^-1^ by 10-fold serial dilutions were employed. Consequently, DNA was extracted as previously reported, to generate a spore standard curve by qPCR by detection of the validated *tfc-1* nuclear gene.

### Oligonucleotide design and quantitative reverse transcription polymerase chain reaction (qRT-PCR)

The primers and hydrolysis probe for the calcineurin catalytic subunit (*cnaA*, NCBI gene bank accession number AGJ95088.1); the Na^+^-K^+^ ATPases *ena1* (ID number 105213), and *ena2* (ID number 113279) DNA sequences were obtained from *M*. *circinelloides* genome database [55]. Design of the primers and hydrolysis probes for each gene was performed using Biosearch Technologies software (www.biosearchtech.com) to ensure the specificity of all detections during qRT-PCR assays (S4 Table). To evaluate the relative gene expression of *gpa*, *pkar1*, and *adh1* from *M*. *circinelloides* protocols described previously were followed [25, 29]. Genomic DNA and total RNA isolation were performed as described previously [18].

### Quantification of intracellular cAMP levels and protein kinase A activity

Spores from MU402, Δ*gpa11*, Δ*gpa12*, Δ*gpa11/* Δ*gpa12*, Δ*cnaA*, Δ*gpa11*+*gpa11wt* or Δ*gpa12*+*gpa12wt* strains were inoculated into YPG media and incubated with constant shaking (150 rpm) for 3 h. Biological samples that were obtained under these conditions were used in either cAMP or PKA measurements.

### cAMP levels

Cells were separated from media and frozen in liquid nitrogen. Upon nitrogen evaporation, 60 mg of biological samples were weighed, and resuspended in 1 mL of 0.1 M HCl, and 250 μL of glass beads (0.1 mm) (BioSpec Products, Inc., Bartlesville, OK, USA) were added. Samples were homogenized by receiving three pulses at full speed for 30 s, with 1 min incubation on ice intermittently, in a beadbeater (607-BioSpec Products, Inc., Bartlesville, OK, United States). The supernatants were used for cAMP measurements using the Direct cAMP ELISA kit (Enzo Life Science, San Diego, USA). Protocols were followed accordingly to the manufacturer’s instructions. The OD was measured at 405 nm on a microplate reader (Bio-Rad Benchmark; Bio-Rad Laboratories, Hercules, CA, USA).

### PKA activity

We obtained crude protein extract (0.5 μg) from each sample, and a PKA-specific kinase activity was quantified by using the PKA Colorimetric Activity Kit (Invitrogen, Carlsbad, CA, United States), as described previously [15].

### Statistical analysis

All data were evaluated by analysis of variance (ANOVA, level of statistically significant difference at α<0.05). Fisher post-hoc test was used.

## Supporting information

S1 Fig The spores from the independent *M*. *circinelloides* knockout strains produced in YPG were observed under **A)** light microscope (100 X), scale bar is equal to 20 μm. **B)** Under scanning electron microscope. Representative photographs from the corresponding strains of *M*. *circinelloides* (1000 X), scale bar is equal to 10 μm.
(DOCX)

S2 Fig Identification of Ena1 and Ena2 from *M*. *circinelloides*. Ena1 from *Hansenulla polymorpha* was used as bait to find Ena homologues in *M*. *circinelloides*. **A)** Clustal W alignment analysis. Identical and similar amino acid residues are shown in black and grey boxes, respectively. **B)** Identity analysis of Ena homologues in *M*. *circinelloides* is shown as identity/similarity in percentage, Mc: *M*. *circinelloides*; Hp: *H*. *polymorpha*.
(DOCX)

S3 Fig Effect of inhibitor of calcineurin FK506 and Triton X-100 on the viability *M*. *circinelloides gpa11* and *gpa12* mutants. **A)** Effect of inhibitor of calcineurin FK506 on the growth of *M*. *circinelloides*. Radial growth of spores grown on YPG or YNB agar plates recorded each day during the experiment. Representative photos show radial growth after 3 days. Three independent experiments were performed for each condition. **B)** 100 spores from each strain were inoculated by spreading on YPG and YNB plates supplemented with 0.005% Triton X-100. Plates were incubated for 1 day. The bars represent the number of colonies formed with treatment versus the colonies formed without treatment. Three independent experiments were performed for each condition. *Statistically significant difference (ANOVA, Fisher, p<0.05).
(DOCX)

S4 Fig Deletion of *gpa genes* in *M*. *circinelloides* confirmed by PCR. **A)** *gpa11*; **B)** *gpa12* and **C)** *gpa11/gpa12* mutation strategy. The 5′ and 3′ regions upstream and downstream from the start and stop translation codons, respectively, were used to flank the *pyrG* (single mutants) or *leuA* (double mutant) selective marker. The diagrams show the recombinant fragments used to delete the *gpa* genes in protoplasts of *M*. *circinelloides* MU402 wild-type strain. The photographs show the molecular confirmation by PCR with specific primers for each gene showing specific PCR-bands that indicate recombination in the corresponding *gpa loci*. **M**: Molecular size markers (kb). **C+**: PCR control for positive amplification (*wt*: 1.1 kb amplicon to identify the wt *gpa11* or *gpa12* genes); Δ*gpa11*: 1.4 kb amplicon to identify the deletion of *gpa11* gene; Δ*gpa12*: 1.3 kb amplicon to identify the deletion of *gpa12* gene; and Δ*gpa11/*Δ*gpa12*: 2.2 kb amplicon to identify the deletion of *gpa11* gene in the mutant Δ*gpa12*.
(DOCX)

S1 Table Sporangiospore mRNA levels of *gpa* genes from *gpa11* and *gpa12* mutant strains from *M*. *circinelloides*. (DOCX)

S2 Table Sporangiospore mRNA levels of *gpa* genes from *cnaA* mutant strain from *M*. *circinelloides*. (DOCX)

S3 Table Oligonucleotides used for PCR assays. (DOCX)

S4 Table Oligonucleotides for qRT-PCR. (DOCX)
