# Supplementary figures and images for: Heterotrimeric G-alpha subunits Gpa11 and Gpa12 define a transduction pathway that control spore size and virulence in Mucor circinelloides
Source: PLoS One. 2019 Dec 30;14(12):e0226682. doi: 10.1371/journal.pone.0226682 (PMC6936849; doi:10.1371/journal.pone.0226682)

**Figure S1**

**
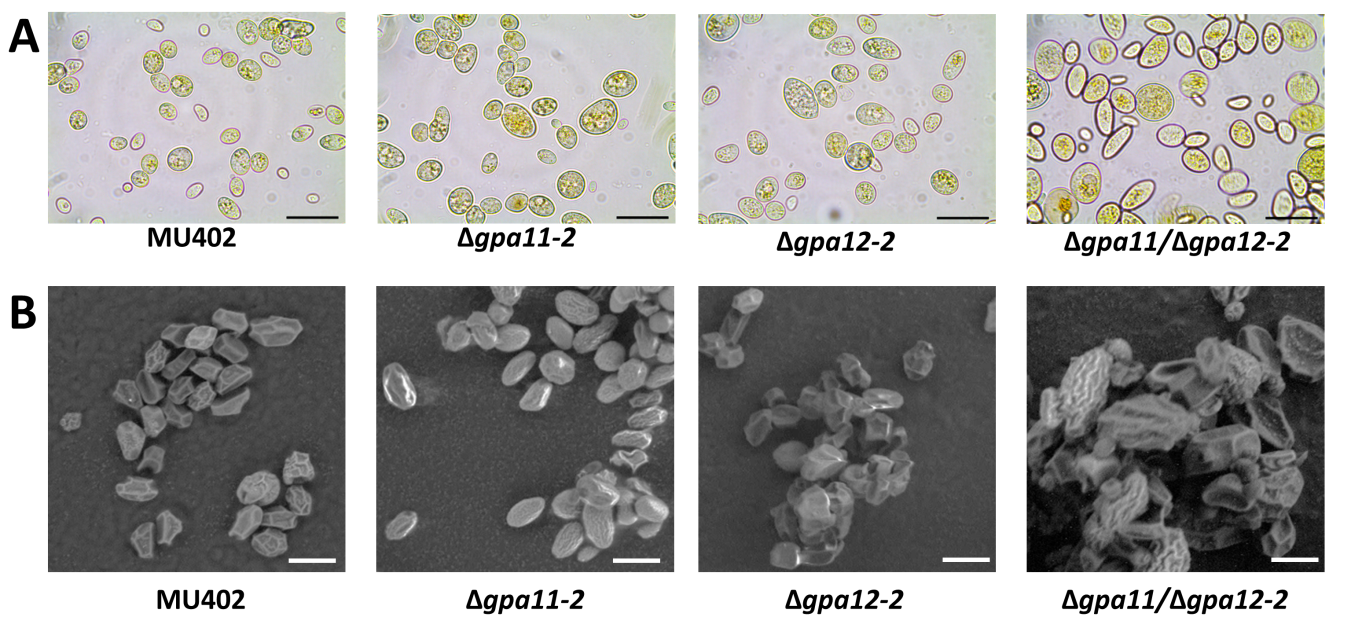
**

Supplement: S1 Fig — The spores from the independent M. circinelloides knockout strains produced in YPG were observed under A) light microscope (100 X), scale bar is equal to 20 μm. B) Under scanning electron microscope. Representative photographs from the corresponding strains of M. circinelloides (1000 X), scale bar is equal to 10 μm. (DOCX) [file pone.0226682.s001.docx]

**Figure S2**

**
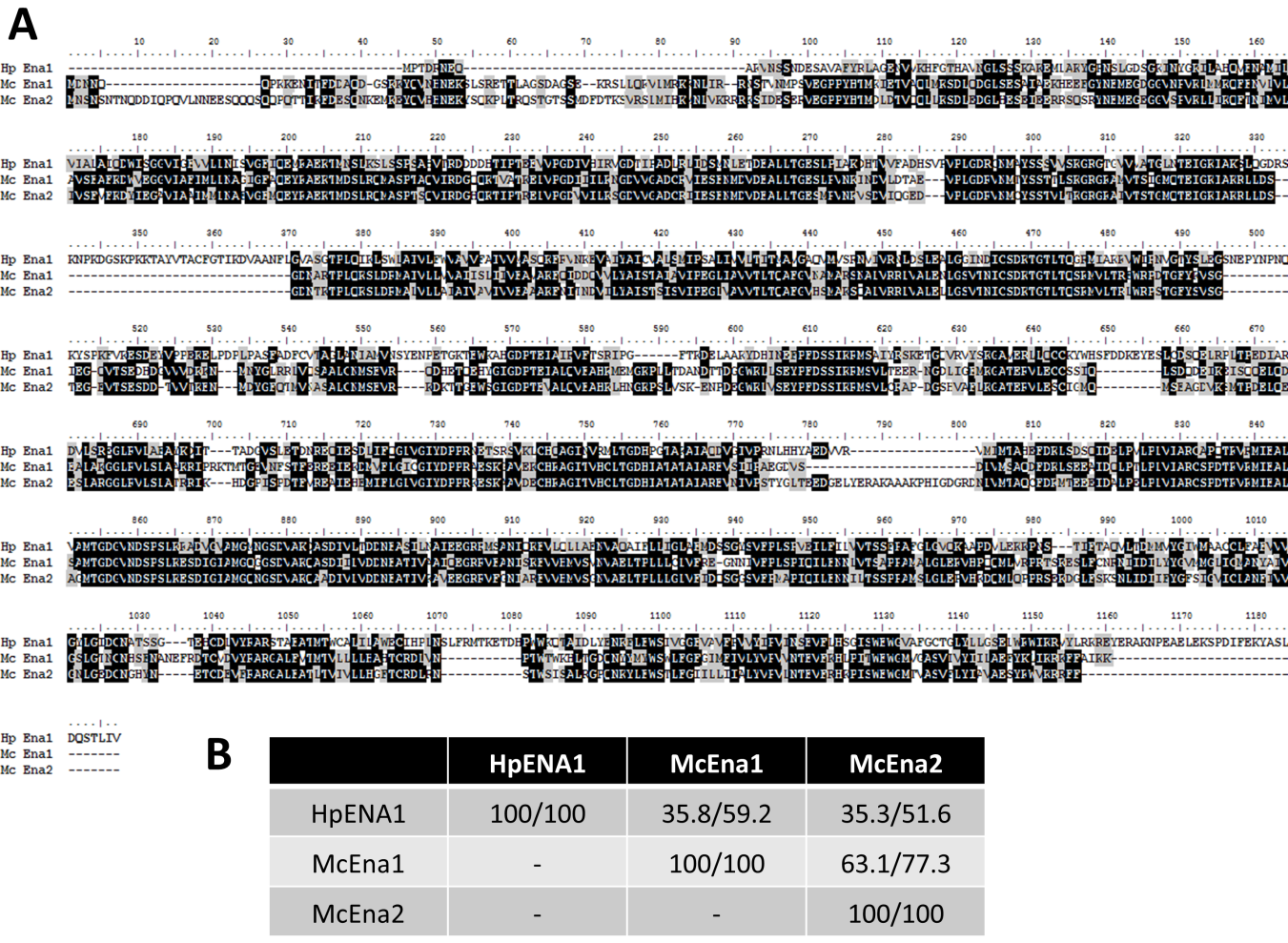
**

Supplement: S2 Fig — Ena1 from Hansenulla polymorpha was used as bait to find Ena homologues in M. circinelloides. A) Clustal W alignment analysis. Identical and similar amino acid residues are shown in black and grey boxes, respectively. B) Identity analysis of Ena homologues in M. circinelloides is shown as identity/similarity in percentage, Mc: M. circinelloides; Hp: H. polymorpha. (DOCX) [file pone.0226682.s002.docx]

**Figure S3**

**A**

**
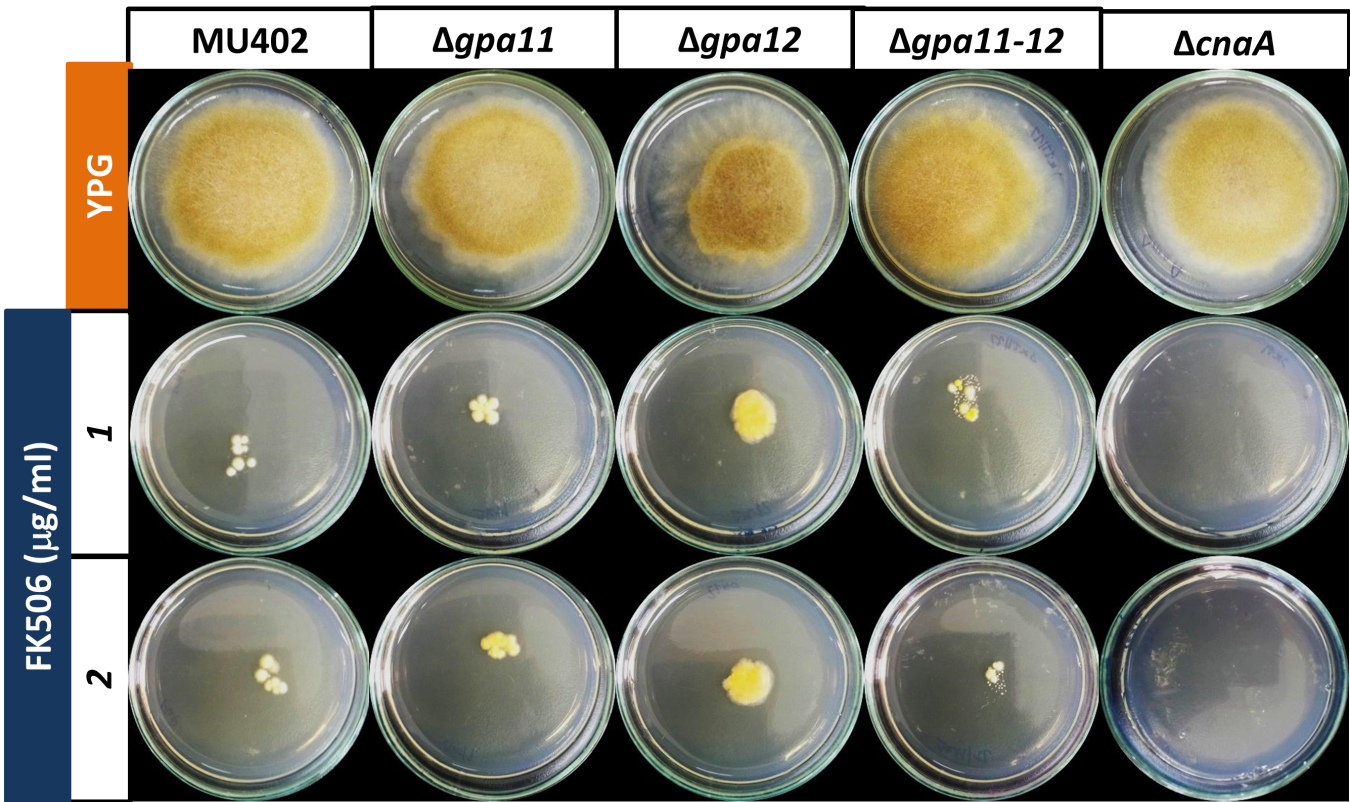
**

**
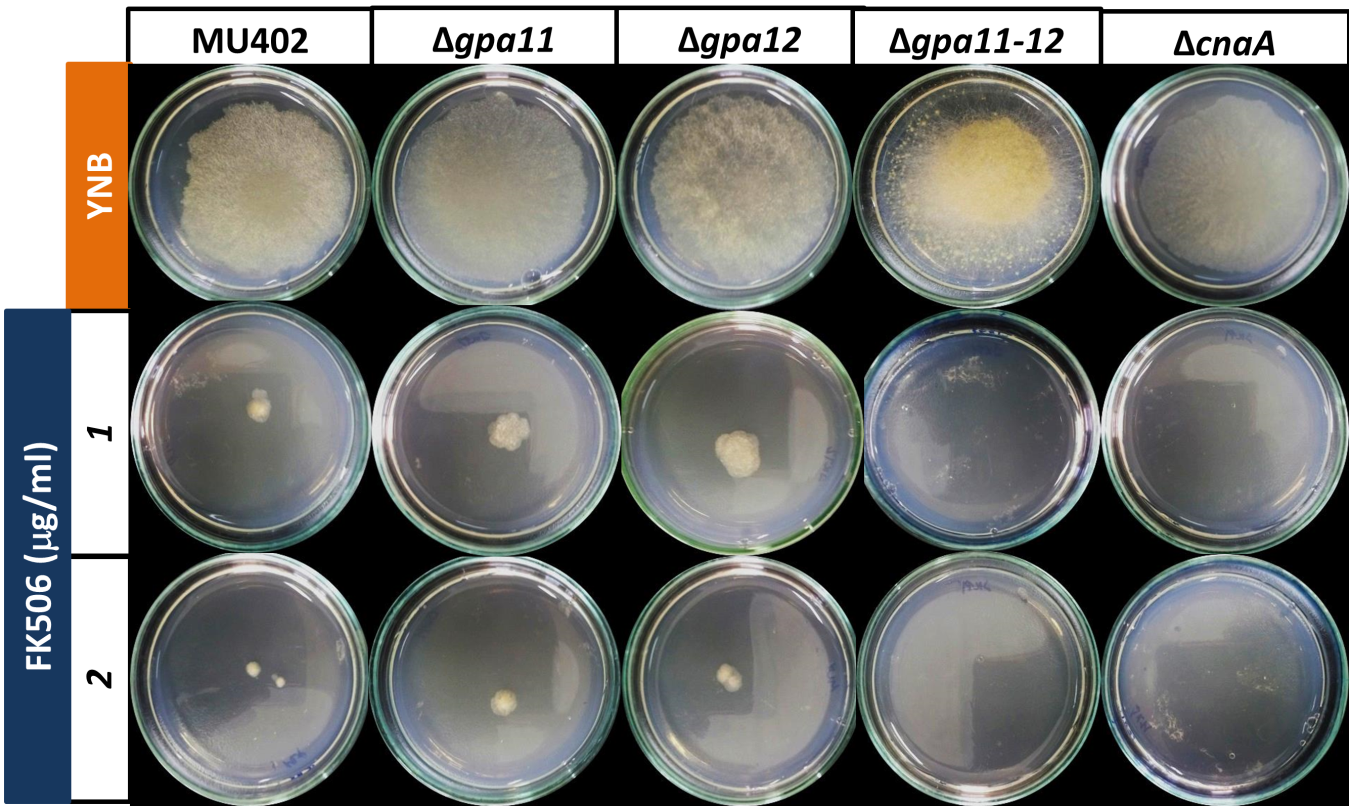
**

**B**

**
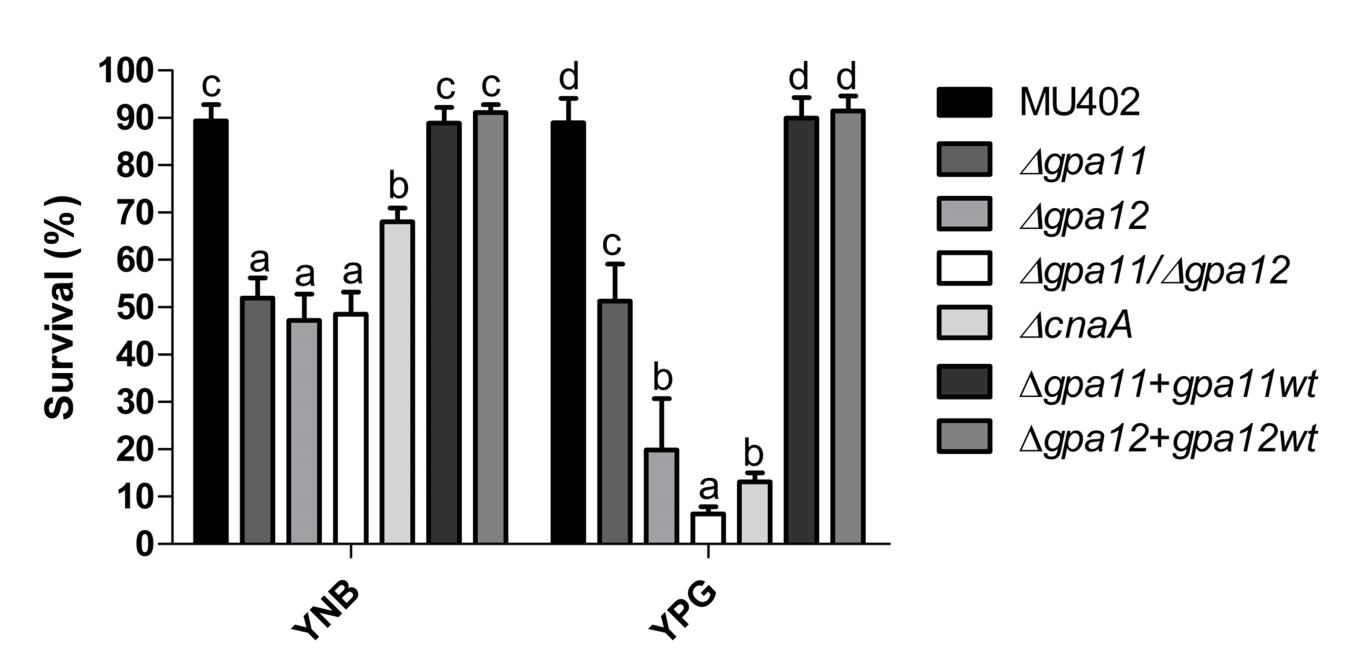
**

Supplement: S3 Fig — A) Effect of inhibitor of calcineurin FK506 on the growth of M. circinelloides. Radial growth of spores grown on YPG or YNB agar plates recorded each day during the experiment. Representative photos show radial growth after 3 days. Three independent experiments were performed for each condition. B) 100 spores from each strain were inoculated by spreading on YPG and YNB plates supplemented with 0.005% Triton X-100. Plates were incubated for 1 day. The bars represent the number of colonies formed with treatment versus the colonies formed without treatment. Three independent experiments were performed for each condition. *Statistically significant difference (ANOVA, Fisher, p<0.05). (DOCX) [file pone.0226682.s003.docx]

**Figure S4**

**
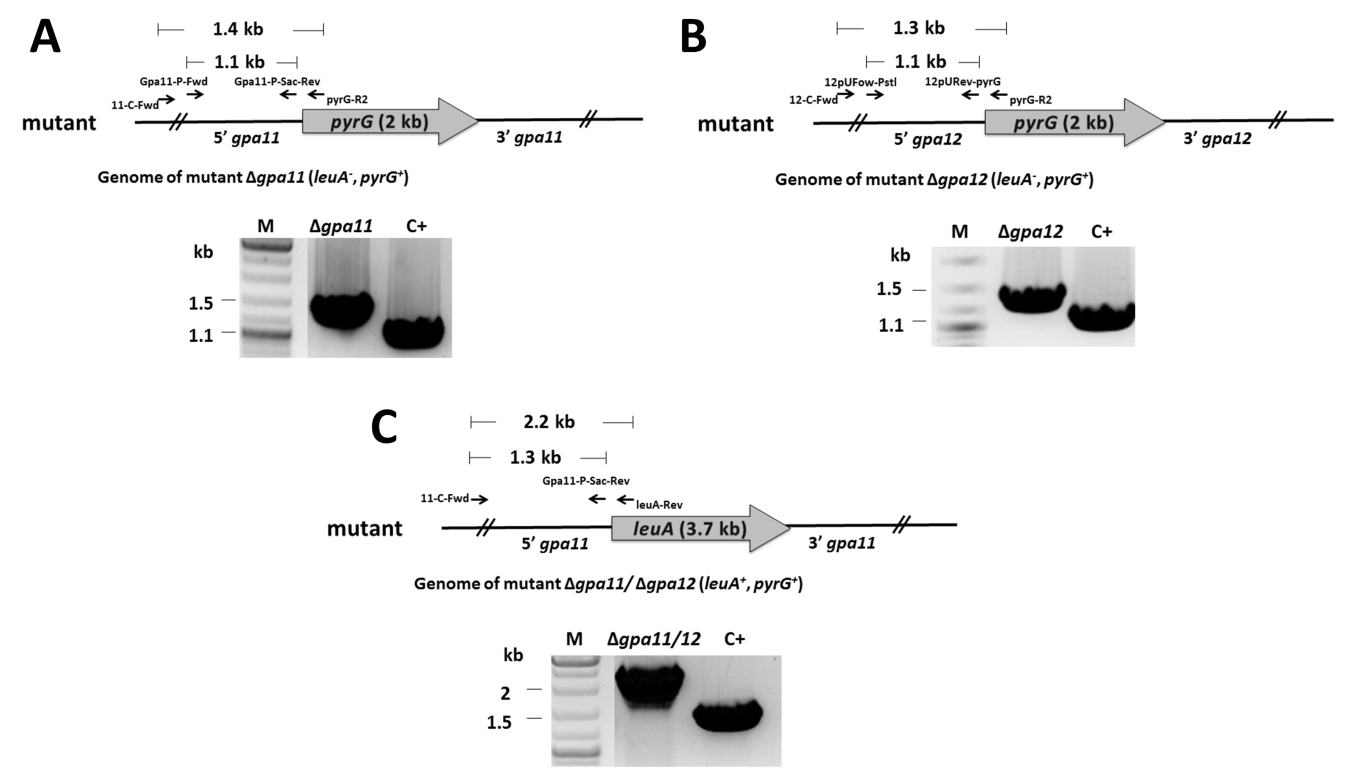
**

Supplement: S4 Fig — A) gpa11; B) gpa12 and C) gpa11/gpa12 mutation strategy. The 5′ and 3′ regions upstream and downstream from the start and stop translation codons, respectively, were used to flank the pyrG (single mutants) or leuA (double mutant) selective marker. The diagrams show the recombinant fragments used to delete the gpa genes in protoplasts of M. circinelloides MU402 wild-type strain. The photographs show the molecular confirmation by PCR with specific primers for each gene showing specific PCR-bands that indicate recombination in the corresponding gpa loci. M: Molecular size markers (kb). C+: PCR control for positive amplification (wt: 1.1 kb amplicon to identify the wt gpa11 or gpa12 genes); Δgpa11: 1.4 kb amplicon to identify the deletion of gpa11 gene; Δgpa12: 1.3 kb amplicon to identify the deletion of gpa12 gene; and Δgpa11/Δgpa12: 2.2 kb amplicon to identify the deletion of gpa11 gene in the mutant Δgpa12. (DOCX) [file pone.0226682.s004.docx]
