# Supplementary material for: Heterotrimeric G-alpha subunits Gpa11 and Gpa12 define a transduction pathway that control spore size and virulence in Mucor circinelloides
Source: PLoS One. 2019 Dec 30;14(12):e0226682. doi: 10.1371/journal.pone.0226682 (PMC6936849; doi:10.1371/journal.pone.0226682)
Supplement: S1 Table — (DOCX) [file pone.0226682.s005.docx]

**Table S1. Sporangiospore mRNA levels of *gpa* genes from *gpa11* and *gpa12***

**mutant strains from *M. circinelloides*.**

|  | **Δ*gpa11*** | **Δ*gpa12*** | **Δ*gpa11/*Δ*gpa12*** |
| --- | --- | --- | --- |
| ***gpa1**** | 1.40 ± 0.35 | 4.14 ± 2.09 | 12.00 ± 5.34 |
| ***gpa2**** | 1.12 ± 0.26 | 0.72 ± 0.38 | 1.30 ± 0.17 |
| ***gpa3**** | 2.87 ± 0.86 | 2.39 ± 0.88 | 6.88 ± 1.27 |
| ***gpa4***** | 2.40 ± 0.89 | 3.62 ± 1.38 | 2.37 ± .038 |
| ***gpa5***** | 1.29 ± 0.13 | 1.36 ± 0.59 | 2.46 ± .016 |
| ***gpa6***** | 2.38 ± 0.12 | 1.15 ± 0.38 | 1.37 ± 0.27 |
| ***gpa7****** | 1.65 ± 0.10 | 1.11 ± 0.04 | 2.16 ± 0.33 |
| ***gpa8**** | 1.23 ± 0.54 | 3.80 ± 1.08 | 5.00 ± 1.5 |
| ***gpa9****** | 2.54 ± 1.1 | 3.44 ± 2.44 | 0.76 ± 0.22 |
| ***gpa10****** | 1 ± 0.11 | 3.17 ± 0.31 | 8.80 ± 2.59 |
| ***gpa11**** | 0 | 1.29 ± 0.12 | 0 |
| ***gpa12****** | 3.31 ± 0.68 | 0 | 0 |

The numbers represent the fold-change in mRNA levels of each gene from corresponding mutant respect to the mRNA levels form wild-type strain.
