# Supplementary material for: Heterotrimeric G-alpha subunits Gpa11 and Gpa12 define a transduction pathway that control spore size and virulence in Mucor circinelloides
Source: PLoS One. 2019 Dec 30;14(12):e0226682. doi: 10.1371/journal.pone.0226682 (PMC6936849; doi:10.1371/journal.pone.0226682)
Supplement: S2 Table — (DOCX) [file pone.0226682.s006.docx]

**Table S2. Sporangiospore mRNA levels of *gpa* genes from *cnaA* mutant**

**strain from *M. circinelloides***

|  | **Δ*cnaA*** |
| --- | --- |
| ***gpa1**** | 2.67 ± 1.21 |
| ***gpa2**** | 3.34 ± 1.51 |
| ***gpa3**** | 2.39 ± 0.59 |
| ***gpa4***** | 1.96 ± 0.74 |
| ***gpa5***** | 1.22 ± 0.24 |
| ***gpa6***** | 0.85 ± 0.24 |
| ***gpa7****** | 1.17 ± 0.19 |
| ***gpa8**** | 3.01 ± 1.99 |
| ***gpa9****** | 0.73 ± 0.16 |
| ***gpa10****** | 1.85 ± 0.89 |
| ***gpa11**** | 0.39 ± 0.08 |
| ***gpa12****** | 0.43 ± 0.16 |

The numbers represent the fold-change in mRNA levels of each gene from corresponding mutant respect to the mRNA levels form wild-type strain.
