# Supplementary material for: Heterotrimeric G-alpha subunits Gpa11 and Gpa12 define a transduction pathway that control spore size and virulence in Mucor circinelloides
Source: PLoS One. 2019 Dec 30;14(12):e0226682. doi: 10.1371/journal.pone.0226682 (PMC6936849; doi:10.1371/journal.pone.0226682)
Supplement: S3 Table — (DOCX) [file pone.0226682.s007.docx]

**Table S3. Oligonucleotides used for PCR assays**

| **Name** | **Secuence 5’-3´** | **Tm(°C)/GC%** |
| --- | --- | --- |
| Gpa11-P-Fwd | CATCGCGTAAACCAAGCTCACATCTCTCTATTG | 68.3/45.5 |
| Gpa11-P-Sac-Rev | TGCAAACAACAACAAgagctcTCACAATAAAAAAGA | 64.6/33.3 |
| Gpa11-T-Sac-Fwd | GgagctcCGTACAGTCAAGAGAGCTTGACATTTGACA | 71.3/48.6 |
| Gpa11-T-Xba-Rev | CtctagaTCCACATCGACAATGATACGATTCTTGGAA | 68.0/40.5 |
| PyrG-Sac-Fwd | GCACCGgagctcTGCCTCAGCATTGGTACTTG | 67.7/53.8 |
| PyrG-Sac-Rev | gagctcGTACACTGGCCATGCTATCG | 69.3/57.6 |
| 12pUFow-Pstl | ATACGctgcagTCTCTCTGCGTAACGAAATGG | 76.9/50 |
| 12pURev-pyrG | **caagtaccaatgctgaggca**TGGTTGCTATGGTGATGATGAT | 82.4/45.2 |
| 12pDFow-pyrG | **cgatagcatggccagtgtac**CCAACAACGCAATCAAACAACC | 85.0/50 |
| 12pDRev-Notl | ATCGgcggccgcAATGATGGAGATGCAGGAGAC | 86.9/60.6 |
| pyrG-R2 | ATCCCACCAGAAGGAGTACATGG | 66.5/52.1 |
| 11-C-Fwd | CCATCTAGGTCTCCTTATTAGGGA | 62.3/45.8 |
| 12-C-Fwd | GGAGAGCGTGCCTAAATCAT | 63.1/50 |
| leuA-Sac-Fwd | GgagctcCTTCTATTATGCAACTCAAGTGCGATGAG | 70.3/47.2 |
| leuA-Sac-Rev | CgagctcCTGCAGTAGCTGTTGATGTTGTTGTTGTATCG | 72.0/48.7 |
| leuA-C-Rev | CCGGCGTCGACAATCATTGGTCAATCTTACCG | 74.5/53.1 |
| gpa11-FWR-XhoI | GGCTActcgagATGGGACAATGTTGCTCTTCCA | 79.49/51.52 |
| gpa11-REV-NotI | TACAgcggccgcAAGATGGCACAATTACAGCAAGCC | 78.59/55.56 |
| gpa12-FWR-SalI | CACTgtcgacCAACCAGCAACGATCTTGTCAG | 74.46/53.15 |
| gpa12-REV-NotI | CACTgcggccgcGTTGTTGGGTAAGAGAAGAAGATT | 77.34/52.78 |

Lowercase letters represent restriction sites

The lowercase and bold letters show the region that hybridizes to the *pyrG* gene
