# Supplementary material for: Heterotrimeric G-alpha subunits Gpa11 and Gpa12 define a transduction pathway that control spore size and virulence in Mucor circinelloides
Source: PLoS One. 2019 Dec 30;14(12):e0226682. doi: 10.1371/journal.pone.0226682 (PMC6936849; doi:10.1371/journal.pone.0226682)
Supplement: S4 Table — (DOCX) [file pone.0226682.s008.docx]

**Table S4.** **Oligonucleotides for qRT-PCR**

| **Gen**  **(ID number*)** | **Forward oligonucleotide (5´-3´)**  **Reverse oligonucleotide (5´-3´)**  **Probe (5´-3´) FAM-BHQ1** | **Tm (°C) / GC (%)** | **Amplification efficiency (%)** | **Amplicon size (bp)** |
| --- | --- | --- | --- | --- |
| mc*cnaA*  (73675) | GCCAAGCGATCAGATTTGGA  GCTGTCAGTCTCCTCTTTCAGTA  AACGAGCGTCTGCCACCTACGC | 66.47 / 50  67.36 / 47.8  74.63 / 63.6 | 94 | 84 |
| mc*ena1* (105213) | GCGCCCTGTTTGTCACCAT  TGACCAAATCACGGCAAGTATG  ACAGTGCTGCTCTTGCTTCATGCC | 69.2 / 57.8  67.1 / 45.4  73 / 54.1 | 92 | 66 |
| mc*ena2*  (113279) | TGCTCGCTGTAGCCCTGAT  CACCAGTCATACCAGCAATCTTC  CCAAGGTAAAGATGATTGAAGCCCTTCA | 68.8 / 57.8  67.1 / 47.8  70.9 / 42.8 | 93 | 80 |
